# Molecular evolution of the proopiomelanocortin system in Barn owl species

**DOI:** 10.1371/journal.pone.0231163

**Published:** 2020-05-05

**Authors:** Karin Löw, Anne-Lyse Ducrest, Luis M. San-Jose, Céline Simon, Vera Uva, Nabil G. Seidah, Antonella Pasquato, Stefan Kunz, Alexandre Roulin

**Affiliations:** 1 Department of Ecology and Evolution, University of Lausanne, Lausanne, Switzerland; 2 Institute of Microbiology, University Hospital Center and University of Lausanne, Lausanne, Switzerland; 3 Laboratory of Biochemical Neuroendocrinology, Clinical Research Institute of Montreal, Montreal, Canada (Affiliated to the University of Montreal); Universite de Rouen, FRANCE

## Abstract

Examination of genetic polymorphisms in outbred wild-living species provides insights into the evolution of complex systems. In higher vertebrates, the proopiomelanocortin (POMC) precursor gives rise to α-, β-, and γ-melanocyte-stimulating hormones (MSH), which are involved in numerous physiological aspects. Genetic defects in POMC are linked to metabolic disorders in humans and animals. In the present study, we undertook an evolutionary genetic approach complemented with biochemistry to investigate the functional consequences of genetic polymorphisms in the POMC system of free-living outbred barn owl species (family Tytonidae) at the molecular level. Our phylogenetic studies revealed a striking correlation between a loss-of-function H9P mutation in the β-MSH receptor-binding motif and an extension of a poly-serine stretch in γ3-MSH to ≥7 residues that arose in the barn owl group 6–8 MYA ago. We found that extension of the poly-serine stretches in the γ-MSH locus affects POMC precursor processing, increasing γ3-MSH production at the expense of γ2-MSH and resulting in an overall reduction of γ-MSH signaling, which may be part of a negative feedback mechanism. Extension of the γ3-MSH poly-serine stretches ≥7 further markedly increases peptide hormone stability in plasma, which is conserved in humans, and is likely relevant to its endocrine function. In sum, our phylogenetic analysis of POMC in wild living owls uncovered a H9P β-MSH mutation subsequent to serine extension in γ3-MSH to 7 residues, which was then followed by further serine extension. The linked MSH mutations highlight the genetic plasticity enabled by the modular design of the POMC gene.

## Introduction

In higher vertebrates, the melanocortin system is involved in numerous physiological aspects such as energy homeostasis, steroidogenesis, exocrine secretion, sexual function, and pigmentation [1–3]. The evolutionarily conserved melanocortin system comprises corticotropin releasing factor (CRF), the proopiomelanocortin (POMC)-derived peptides α-, β-, and γ-melanocyte-stimulating hormones (MSH), adrenocorticotropic hormone (ACTH), the G protein-coupled seven-transmembrane melanocortin receptors (MC1-5R), as well as the melanocortin antagonists agouti and agouti-related protein. The POMC precursor displays a modular organization that is highly conserved among tetrapods. Processing of POMC through cleavage at dibasic motifs primarily occurs in the pituitary, where proprotein convertase (PC)1/3-expressing corticotropic cells produce pro-γ-MSH, ACTH and β-lipotropin (β-LPH). PC1/3- and PC2-expressing melanotropic cells further process the PC1/3 cleavage products to α-, β-, γ3-and γ2-MSH, as well as corticotropin-like intermediate peptide (CLIP), γ-lipotropin (γ-LPH) and β-endorphin (β-end) [4, 5]. The α-, β-, and γ-MSH forms are highly conserved between species, share a common receptor-binding motif HFRW, and can signal with varying efficacy through MC1R, MC3R, MC4R and MC5R, whereas MC2R can only be activated by ACTH [3]. Pituitary cleavage products are released into the bloodstream and delivered to peripheral tissues, where they activate MCR. In the brain, sequential POMC processing by PC1/3 and PC2 occurs in neurons of the hypothalamus and brain stem, leading to the release of MSH peptides, which act as neuromodulators in autocrine and paracrine manner.

POMC expressing neurons in the arcuate nucleus of the hypothalamus are central to energy homeostasis. Release of MSH peptides in their target projection area, the paraventricular nucleus, leads to activation of post-synaptic MC4R, which ultimately results in a reduction of food-intake [6–8]. POMC and MSH deficiencies, as well as MC4R deficiencies/mutations or pharmacological blockade of MC4R have been linked to hyperphagia and obesity, increased lipid up-take, triglyceride synthesis and fat accumulation in white adipose tissue in animals and humans [9–16].

Over the past 15 years, we performed an in-depth genetic analysis of the melanocortin system in a wild-living, outbred population of the European barn owl *Tyto alba alba* (TA) in Switzerland. *Tyto alba alba* is a very interesting species from an evolutionary ecology point of view: it is cosmopolitan, efficiently reproduces with several breeding attempts per year, displays highly variable coloration phenotypes and shows complex social behavior, which make it particularly suitable to analyze covariation of melanocortin linked phenotypes [1, 17]. When sequencing the POMC gene in a Swiss population of TA we now discovered that all tested individuals carried a homozygous H9P mutation in β-MSH that changed its receptor-binding site from HFRW to PFRW, likely impeding its function. Rather unexpected, affected animals showed no overt phenotype, specifically, no signs and symptoms of obesity, despite the fact that *POMC* mutations affecting production and/or function of β-MSH are associated with obesity in human [11–15]. Interestingly, the β-MSH H9P mutation in TA is associated with a remarkable polymorphism in the γ3-MSH locus, namely a microsatellite sequence of 10–24 AGC repeats, translating into poly-serine stretches. Intrigued by the apparent association between the β-MSH H9P mutation and the polymorphism in the γ3-MSH locus, we established a phylogenetic tree for Tytonidae species with a worldwide distribution [18], and determined their *POMC* sequences. We were able to trace the origins of the two *POMC* polymorphisms and provide evidence for a genetic link. At the functional level, we demonstrate that the β-MSH H9P mutation results in loss of receptor signaling. Extension of the poly-serine stretches in the γ-MSH locus lead to an increase in γ3-MSH levels at the expense of γ2-MSH, reducing the overall γ-MSH signaling, possibly as part of a negative feedback mechanism. Extension of the γ3-MSH poly-serine stretches ≥7 further markedly increases peptide hormone stability in plasma, likely affecting its endocrine function.

## Material and methods

### Synthetic MSH peptides used in MCR signaling assays

SA β-MSH H9: AGGSYRMR**HFRW**HAPLKD (GenScript USA Inc., Piscataway, New Jersey, USA); SA β-MSH P9: AGGSYRMR**PFRW**HAPLKD (GenScript USA Inc.); TA β-MSH P9: AGGSHRVR**PFRW**HAPLKD (GenScript USA Inc.); TA β-MSH H9: AGGSHRVR**HFRW**HAPLKD (GenScript USA Inc.); TA β-MSH P9, Y5: AGGSYRVR**PFRW**HAPLKD (GenScript USA Inc.); TA β-MSH P9, M7: AGGSHRMR**PFRW**HAPLKD (GenScript USA Inc.); SA / TA γ2-MSH: YVMSHFRWNKFG, C-terminus amidated (GenScript USA Inc.); SA γ3-MSH 3 S: YVMSHFRWNKFGRRNSSSGGGGGH (GenScript USA Inc.); TA γ3-MSH 18S: YVMSHFRWNKFGRRNSSSSSSSSSSSSSSSSSSGGH (GenScript USA Inc.); TA γ3-MSH 7 S: YVMSHFRWNKFGRRNSSSSSSSGGH (Top-peptide Co., Ltd, Shanghai, China); TA γ3-MSH 11 S: YVMSHFRWNKFGRRNSSSSSSSSSSSGGH (Top-peptide Co., Ltd); TA γ3-MSH 13 S: YVMSHFRWNKFGRRNSSSSSSSSSSSSSGGH (Top-peptide Co., Ltd, Shanghai, China); TA γ3-MSH 19S: YVMSHFRWNKFGRRNSSSSSSSSSSSSSSSSSSSGGH (Top-peptide Co., Ltd); TA γ3-MSH 5 S: YVMSHFRWNKFGRRNSSSSSGGH (SynPeptide CO., LTD, Shanghai, China); TA γ3-MSH 10 S: YVMSHFRWNKFGRRNSSSSSSSSSSGGH (SynPeptide CO., LTD); human β-MSH H9 (wt): DEGPYRMEHFRWGSPPKD-NH2 (Top-peptide Co., Ltd, Shanghai, China); human β-MSH P9: DEGPYRMEPFRWGSPPKD-NH2 (Top-peptide Co., Ltd, Shanghai, China); human β-MSH C5: DEGPCRMEHFRWGSPPKD-NH2 (Top-peptide Co., Ltd, Shanghai, China); human β-MSH L15: DEGPYRMEHFRWGSLPKD-NH2 (Top-peptide Co., Ltd, Shanghai, China); human γ2-MSH: YVMGHFRWDRFG-NH2 (Top-peptide Co., Ltd, Shanghai, China); human γ3-MSH (wt): YVMGHFRWDRFGRRNSSSSGSSGAGQ (Top-peptide Co., Ltd, Shanghai, China); human γ3-MSH SSG SSG: YVMGHFRWDRFGRRNSSSSGSSGSSGSSGAGQ (Top-peptide Co., Ltd, Shanghai, China); human γ3-MSH SSG: YVMGHFRWDRFGRRNSSSSGSSGSSGAGQ (SynPeptide CO., LTD, Shanghai, China). All peptides were delivered in lyophilized form and had ≥ 98% purity. After resuspension of synthetic peptides in H_2_O, exact MSH concentrations were determined in stock solutions by UV-adsorption.

### Reconstruction of the phylogenetic tree of Tytonidae

Genomic DNA was extracted from blood, feathers and footpads of Tytonidae kindly donated by different museum collections around the world and from blood of wild populations of SA and TA using the DNAeasy Blood and Tissue kit (Qiagen, Hombrechtikon, Switzerland). The phylogenetic analysis was based on a subset of 34 owl taxa, five mitochondrial genes (a total length of 2796 bp comprising cytochrome oxidase 1, 16S rRNA, control region, cytochrome B, and mitochondrially encoded NADH:ubiquinone oxidoreductase core subunit 6), and two nuclear genes (a total length of 1497 bp comprising c-MOS and RAG1) [18]. Preceding the phylogenetic analysis, best-fit substitution models were estimated for each gene, with MrModeltest 2.0 [19], using the Akaike information criterion [20]. The molecular phylogeny was computed using Bayesian Inference (BI) with BEAST v.1.8.4 [21]. Divergence times were estimated based on prior knowledge about the split age (ca. 45 mya) between Tytonidae and Strigidae *(Strix aluco SA)*, taken from a study that used a large sampling of birds and fossil calibrations to date the phylogeny [22]. The analysis was run under an uncorrelated relaxed lognormal clock model, and a birth-and-death tree model. A mean substitution rate of 0.016 was used for mitochondrial DNA, and 0.00135 for nuclear DNA [23]. Standard deviations were set as 0.005 and 0.0005 for mitochondrial and nuclear DNA, respectively. A normal distribution prior was set on the root of the tree, with a mean of 45 and standard deviation of 2.5. Improper default priors (1/x) were replaced by proper weakly informative priors (exponential distribution with mean 1). The MCMC chain was run for 200 million generations sampled every 20.000 generations. Tracer v1.6.0 [24] was used to confirm chain convergence and effective sample size (ESS) values of all parameters. The maximum credibility tree and the 95% highest posterior density (95% HPD) distributions for all tree nodes were generated on TreeAnnotator v1.8.2 [21], with a burn-in of 10% of the trees. The final was visualised on FigTree v1.4.2 (http://tree.bio.ed.ac.uk/software/figtree).

### Genotyping of *γ*3-MSH and β-MSH loci in *POMC* of Tytonidae

Genomic DNA from Tytonidae blood, feathers or footpads was used to identify γ-MSH and β-MSH sequences after PCR amplification of the corresponding *POMC* fragments. For the Swiss (*Tyto alba alba*) and Israeli (*Tyto alba erlangeri*) populations, we collected blood samples or feathers during the breeding season and genotyped all individuals followed between 1999 and 2018 (n = 5852), and 2009 and 2012 (n = 1439), respectively [25]. For blood drawing, we punctured the brachial vein, collected 50 μl blood samples with heparinized capillaries, and immediately stored samples on dry ice for transport. For long term storage, samples were kept at -80°C until DNA extraction.

A 500 bp fragment was PCR amplified within exon 3 of the *POMC* gene covering the γ-MSH, ACTH and β-MSH loci. The amplicons of each individual were purified with MinElute PCR purification kit (Qiagen, Hombrechtikon, Switzerland). PCR products were then directly sequenced (Microsynth Balgach, Switzerland) or, if heterozygous for γ3-MSH, TA-cloned in pGEMT (Promega, Duebendorf, Switzerland) prior to sequencing. Sequences were aligned with CodonCode Aligner 3.7.1.2 (CodonCode Corporations, Dedham, MA, USA) and the number of serine repeats in γ3-MSH determined. If the material was of low quality (e.g., old museum footpads), γ3-MSH and β-MSH spanning fragments were separately amplified and sequenced. Once the serine repeats were characterized for the different taxa, additional individuals of *Tyto alba (T*. *a*.*) alba* (Europe), *T*. *a*. *affinis* (Africa), *T*. *a*. *erlangeri* (Middle East), *T*. *javanica javanica* (south-east Asia), *T*. *j*. *delicatula* (Australia) and *T*. *furcata pratincola* (North America) were genotyped to determine their precise poly-serine microsatellite length. To this end, the γ3-MSH locus was amplified with fluorescin labeled primers and PCR fragments were analyzed with an ABI 3100 sequencer using the GeneScan^TM^ ROX 350^TM^ size standard. Serine repeat lengths were assigned by GENEMAPPER 4.0 (Life Technologies, Thermo Fisher Scientific Schweiz AG, Reinach, Switzerland). Genbank accession numbers for the *POMC* sequences of the individual taxa are given in S1 Table. S3 Table provides information on the museum samples used in this study for POMC genotyping.

### *POMC*, *PCSK1* and *PCSK2* gene expression analysis in different tissues of *Tyto alba alba*

Bursa, liver, heart, intestine, thoracic muscle, testis, growing back and breast feathers, pituitary gland, hypothalamus, cortex and cerebellum were sampled from a 57-day-old nestling barn owl (TA) that had died in its nest for unknown reasons. Dissected tissue was immediately transferred to dry ice and then kept at -80°C. Between 20 and 120 mg of the different tissue sample were bead-homogenized at 4°C with Trizol (Life Technologies) in a MagNA Lyser (Hoffmann-La Roche Ltd., Basel, Switzerland) at 6500 rpm for 3x 30 sec. Total RNA was extracted from tissue homogenates using the RNeasy kit (Qiagen) and 5 μl aliquots were diverted to assess quantity and quality with a Qubit fluorometer (Life technologies) and Fragment analyzer (Advanced analytical, Labgene, Switzerland). Only total RNA samples with a RQN > 9.0 were used to quantify gene expression. The expression of *POMC*, *PCSK1* and *PCSK2* genes was measured by RT-qPCR using Elongation factor 1A (*EEF1A*, KU712278) and ribosomal protein L13 (*RPL13*, KU712291) as reference genes as previously described [26]. Amplification efficiencies for *POMC*, *PCSK1* and *PCSK2* cDNA were 98.5%, 100% and 104%.

### Identification of cDNA sequences coding for *Tyto alba alba POMC*, *PC1/3*, *PC2*, *MC3R* and *MC4R*, and for *Strix aluco POMC*

Total RNA was isolated from the brain extract of the previously described nestling barn owl (TA), using RNeasy kit (Qiagen). One μg total RNA was subjected to RNA-ligase-mediated (RLM) oligo capping (GeneRacer kit, Life Technologies) to then selectively reverse-transcribe the full length mRNA transcripts using 200 U/μl SuperScript^™^ III RT and 2.5 μM GeneRacer^™^ Oligo dT Primer in a 20 μl reaction. After RNase H treatment, 10% of the produced RACEready first-strand cDNA were used to amplify the desired TA cDNA fragments in a polymerase chain reaction (PCR) with a combination of gene specific primers and GeneRacer^TM^ 5’ and 3’ primers. After subcloning, sequences were determined and aligned to yield full length coding sequences (CDS). Sequences for TA *PCSK1* and *PCSK2* mRNA are published under genbank accession numbers KU712308-9 and KU712310, respectively (San-Jose et al., 2017). The TA full length *POMC* CDS spreading over exons 2 and 3 was inferred from the genomic sequence in combination with 5’RACE on brain RNA using POMC specific primers (San-Jose et al., 2017, genbank accession number KU712269.1). The TA *MC3R* and *MC4R* one exon genes could be sequenced from genomic DNA with a genome walking approach (GenomeWalker universal kit, Clontech, Takara Bio Europe/Clontech, Saint-Germain-en-Laye, France). The following external primers were used to determine their sequences: MC3R_239Fw: 5’-GAA GAG CAG CCT GTG ACT GAC AAA AA-3’, MC3R_1273Rev: 5’-GTA GCA TTC CCT TTC CGG ATT CAT TT-3’ and MC4R_584Fw: 5’-GCT CGT GCT GCA TCT GAA TCT ACT GT-3’, MC4R_1057Rev: 5’-AGG ATC TGA AGG CTT GGA AGT CTG TG-3’. The newly identified mRNA sequences for TA *MC3R* and *MC4R* are registered under accession numbers KY189305 and KY189307-8, respectively. The SA *POMC* mRNA sequence was determined with analogous methods as described [27] (Accession number: KF201581).

### Cloning of *Tyto alba alba MC3R*, *MC4R*, *PCSK1* and *PCSK2*

*Tyto alba alba MC3R*, *MC4R*, *PCSK1* and *PCSK2* were PCR amplified from barn owl reverse-transcribed total RNA thereby adding a V5-tag to the N-terminus of both *MCR* isoforms and a myc-tag to the C-terminus of both *PCSK* isoforms. PCR products were inserted into expression vector pcDNA 3.1 IntA containing the human cytomegalovirus (CMV) promoter (kindly provided by Dr. Robert Garry, Tulane University). Untagged human MC3R (genbank accession number NM_019888) and MC4R (genbank accession number NM_005912) were expressed from plasmid pCMV6-XL4 (OriGene Technologies, Inc., Rockville, Maryland, USA).

### Synthesis and cloning of *Tyto alba alba* (TA) and *Strix aluco* (SA) *POMC*

The different TA *POMC* cDNA variants with 3, 5, 7, 10 and 18 serine residues and the SA cDNA variants with 3 and 18 serine residues were commercially synthesized (GenScript USA Inc., Piscataway, New Jersey, USA) according to the cDNA sequence information obtained from the above analyses. A Kozak sequence was added upstream of the start codon, an HA tag (YPYDVPDYA) inserted downstream of the signal peptides MPLWSSLPVVLGLLLWHLAGASGP and MASALWGSLPVVLGLLLWHPAGASGP in SA and TA *POMC*, respectively, and a V5 tag (GKPIPNPLLGLDST) was added to the C-terminus upstream of the stop codon. *POMC* variants with differing numbers of serine repeats were produced by introduction of the according numbers of AGC codons in the γ3-MSH locus. HA- and V5-tagged *POMC* cDNAs were cloned into the expression vector pcDNA 3.1 IntA.

### Cloning of *Tyto alba alba* (TA) N-terminal POMC ladder fragments

The pcDNA 3.1 IntA plasmids coding for N-terminally HA-tagged TA full length *POMC* with 3, 5, 7, 10 or 18 serine residues (see above) served as templates for PCR-amplification of the N-terminally HA-tagged TA *POMC* cleavage site 3 fragments with 3, 5, 7, 10 and 18 serine residues, respectively, and also for amplification of N-terminally HA-tagged *POMC* cleavage site 2 fragment. PCR products thus contained from N- to C-terminus: the *POMC* signal peptide, the HA tag, and part of the mature *POMC* sequence comprising the N-terminal fragment and γ3-MSH in case of cleavage site 3 fragments, and γ2-MSH in case of cleavage site 2 fragment, followed by the dibasic cleavage motifs KR and RR, respectively. All PCR products were cloned into pcDNA 3.1 IntA.

### Cloning of *Strix aluco* (SA) N-terminal POMC ladder fragments

SA N-terminal *POMC* ladder fragments with the wild-type sequence were produced from N-terminally HA-tagged SA full length *POMC* template carrying 3 serine residues in the γ3-MSH locus. SA HA-tagged *POMC* ladder fragments ended with the dibasic motif RK at cleavage site 1, RR at cleavage site 2, or KR at cleavage site 3 (3 serine residues in γ3-MSH locus) and thus behind the POMC N-terminal fragment, γ2-MSH, or γ3-MSH, respectively. The SA N-terminal *POMC* ladder fragments, which contained the amino acids EE instead of RK at cleavage site 1 to prevent cleavage by endogenous basic PCs, were produced by PCR from a point mutated N-terminally HA-tagged SA full length *POMC* template in analogy to the wild-type versions. All PCR products were cloned into pcDNA 3.1 IntA.

### *In Vitro* characterization of *Tyto alba alba* (TA) PC1/3 and PC2 activity

Human embryonic kidney cells containing the SV40 T-antigen (HEK293T) were obtained from the American Type Culture Collection (ATCC, CRL-3216^TM^) and were grown in DMEM GlutaMax^TM^ medium (4.5 g/l D-Glucose, Gibco, Life Technologies) containing 10% Fetal Calf Serum (FCS), 100 units/ml Penicillin and 100 μg/ml Streptomycin, unless otherwise indicated.

HEK293T cells were calcium-phosphate transfected in triplicate with TA *PCSK1* or *PCSK2*-plasmid, in combination with either *mouse 7B2*- or *IRES GFP*-plasmid (1 μg of each of the two plasmids per 3.8 cm^2^ well). Supernatants of mock-transfected cells (*IRES GFP*-plasmid only) served as negative controls. Supernatants were harvested 56 h post transfection for the *in vitro* assay and CaCl_2_ was added to a final concentration of 5 mM. The pH was adjusted to pH 5.5 with 88 mM acetate buffer. In case of investigation of the pH dependence of the two PCs, the pH was adjusted to values between pH 4.1 and pH 8.1. Calcium dependence was analyzed in the presence of 10 mM EDTA. The fluorogenic substrate Pyr-ERTKR-AMC (L-pyroglutamyl-(arginine-threonine-lysine-arginine)-7-amino-3-methylcoumarin) was added to a final concentration of 10 μM. Cleavage induced fluorescence by the liberated AMC unit was measured with a TriStar LB 941 reader using MikroWin 2000 software (Berthold Technologies, Switzerland). The slope in the phase of linear fluorescence increase (relative fluorescence units (RFU)/min) was used as a read out for cleavage activity of the two basic PCs.

### Analysis of *Tyto alba alba* (TA) and *Strix aluco* (SA) POMC cleavage by *Tyto alba alba* PC1/3 and PC2

HEK293T cells were transfected with SA or TA *POMC* alone, or in combination with either *mouse 7B2* [28], *mouse 7B2 and* TA *PCSK1*, or *mouse 7B2*, TA *PCSK1* and TA *PCSK2*. Plasmid mixes contained 1 μg each of the above plasmids, and were supplemented to a total of 4 μg DNA with *IRES GFP* plasmid for calcium-phosphate transfection, or contained 0.5 μg plasmid each, and were supplemented to a total of 2 μg DNA with *IRES GFP* plasmid for Lipofectamine 3000 transfection in 3.8 cm^2^ wells. SA POMC ladder fragments used to unambiguously identify bands in PC-cleaved SA POMC were calcium phosphate transfected at 1 μg/well in combination with *mouse 7B2* plasmid (1 μg/well). Supernatants (1 ml/well) were harvested 56 h post transfection for POMC cleavage product identification, and aliquots of supernatant were collected between 28 h and 104 h post transfection to establish cleavage kinetics. Supernatants were supplemented with dissolved Complete protease inhibitor mix (Complete^TM^, Mini Protease Inhibitor Cocktail, F. Hoffmann-La Roche Ltd., Basel, Switzerland) and 6x reducing Laemmli buffer (12% (w/v) SDS, 47% (v/v) glycerol, 0.06% (w/v) bromphenol-blue, 600 mM DTT, 60 mM Tris-HCl, pH 6.8) to final concentrations of 1x. Samples were heated for 10 min to 98°C and frozen at -20°C.

For cell lysate preparation, layers of transfected HEK293T cells were washed with PBS, and 250 μl ice cold CelLytic^TM^ M buffer (Sigma-Aldrich GmbH, Buchs, Switzerland) containing 1x protease inhibitor mix were added per well. Cells were lysed under agitation for 30 min at 4°C, cell slurry was transferred to Eppendorf tubes and cleared by 30 min centrifugation at 14000 g. 6x reducing Laemmli buffer was added to cleared lysates to a final concentration of 1x, samples were heated for 10 min to 98°C and frozen at -20°C.

For Western blot analysis 10 μl to 30 μl samples of cell lysate and supernatant were separated on 10%, 12% or 18% pre-cast Novex^TM^ Tris-Glycine protein gels (Thermo Fisher Scientific AG, Reinach, Switzerland) and were transferred to 0.45 μm pore-size nitrocellulose membranes. Full-length POMC and N-terminal POMC ladder fragments were detected via the N-terminal HA tag with rat monoclonal (clone 3F10) anti-HA high affinity antibody (IgG1, 33 ng/ml, Roche Diagnostics GmbH, Mannheim, Germany), in combination with peroxidase-conjugated polyclonal rabbit anti-rat purified immunoglobulin fraction (433 ng/ml, Dako Schweiz AG, Baar, Switzerland). PC1/3 and PC2 were detected via their C-terminal myc tag with polyclonal rabbit anti-myc tag antibody (1: 1000, Cell Signaling Technology^R^, BioConcept, Allschwil, Switzerland) and HRP-coupled polyclonal goat anti-rabbit IgG (1:3000, Santa Cruz Biotechnology Inc., Heidelberg, Germany). Tubulin was used as a loading control in cell lysates and was detected with mouse monoclonal (clone B-5-1-2) anti α-Tubulin IgG1 (1:3000, Sigma-Aldrich Chemie GmbH) in combination with peroxidase-conjugated polyclonal rabbit anti-mouse purified immunoglobulin fraction (433 ng/ml, Dako Schweiz AG). The signal was developed in a chemiluminescence reaction with the enhanced sensitivity Advansta Western Bright^TM^ Siruius kit (Witec AG, Luzern, Switzerland). Blots were scanned with a luminescence image analyzer (Image Quant Las 4000 mini) using the Image Quant Las 4000 program, version 1.2 (GE Healthcare AG, Glattbrugg, Switzerland).

### Densitometric quantification of steady state levels of N-terminal cleavage site 3 product in Western blot

16 bit gray scale Western blot images with a suitable exposure were selected and bands densitometrically quantified using ImageJ software (version 1.45s, Wayne Rasband, National Institutes of Health, USA, public domain). For analysis of the production efficiency of POMC N-terminal cleavage site 3 fragment, the bands representing N-terminal cleavage site 5 and 3 products were jointly outlined (separately for each lane). For evaluation of the degradation efficiency, bands representing N-terminal cleavage site 3 and 2 products were simultaneously outlined. The density profiles were separately plotted for the two band combinations. The two peaks corresponding to either band 5 and 3, or band 3 and 2 were delineated and pixel numbers determined in the area underneath the corresponding peaks. Pixel numbers were then expressed as the percentage of total pixels measured for the two peaks together. In this way, the relative pixel density of the two band pairs was determined. The relative pixel density of band 3 was divided by the relative pixel density of band 5 as a measure for the production efficiency, and the relative pixel density of band 2 was divided by the relative pixel density of band 3, as a measure for the degradation efficiency. The production efficiency was subsequently divided by the degradation efficiency for each TA POMC variant with a defined number of serine residues and was normalized within the same blot to the quotient of production and degradation efficiencies of TA POMC 3 S from the corresponding time point in the expression kinetic.

### Determination of the glycosylation status of *Tyto alba alba* (TA) and *Strix aluco* (SA) POMC precursor

HEK293T cells were calcium phosphate transfected in 3.8 cm^2^ wells with 1 μg HA-tagged TA *POMC 18 S—*or SA *POMC 3 S* plasmid, each in combination with 1 μg mouse *7B2* plasmid. Supernatants were removed 49 h after transfection, cells were washed with PBS and cell lysates were harvested in 250 μl ice cold CelLytic^TM^ M buffer (Sigma-Aldrich GmbH) containing 1x Complete^TM^ protease inhibitor mix (F. Hoffmann-La Roche Ltd.). Lysates were cleared by 30 min centrifugation at 14000 g and were then subjected to Endoglycosidase H (Endo H) or Peptide:N-Glycosidase F (PNGase F) digests (New England Biolabs Inc., Bioconcept). To this end, 10 μl of 10 x glycoprotein denaturing buffer (5% SDS, 400 mM DTT) were added to 90 μl of cleared lysate, and samples were boiled for 10 min. Denatured lysates were then split into four 20 μl aliquots and components required for Endo H or PNGase F treatment were added to yield final digest volumes of 30 μl. Denatured lysates were also mock treated with buffers used for Endo H and PNGase F digests with no enzymes added. The final EndoH digestion reaction contained the 1.7 fold diluted lysate in 2.67 mM Tris-HCl, 6.67 mM NaCl, 0.67 mM EDTA, 0.33% SDS, 26.7 mM DTT, 50 mM sodium citrate, pH 5.5 with 66.7 units/μl Endo H. The final PNGase F digestion reaction consisted of the 1.7 fold diluted lysate in 2 mM Tris-HCl, 5 mM NaCl, 0.5 mM EDTA, 5% glycerol, 1% NP-40, 0.33% SDS, 26.7 mM DTT, 50 mM sodium phosphate, pH 7.5 with 50 units/μl PNGase F. For deglycosylation, samples were incubated for 2 h at 37°C. Deglycosylation was terminated by the addition of 5 μl 6x reducing Laemmli buffer and boiling for 10 min. Mock-, Endo H and PNGase F treated lysates from cells over-expressing TA or SA POMC were separated on 12% Tris-glycine gels and deglycosylation patterns were analyzed in Western blot via the N-terminal HA tag.

### Measurement of cAMP-signaling activities of synthetic β-MSH and γ-MSH ligands on *Tyto alba alba* (TA) and *Human* MC3R and MC4R

5 x 10^4^ HEK293T cells were seeded per well in 100 μl medium in Costar 96 well clear bottom plates (Corning Inc., New York, USA) and were transfected (lipofectamine 3000) with 1 ng TA or *Human MCR* plasmid or *IRES GFP* control plasmid in combination with 100 ng pGloSensorTM-20F cAMP plasmid (E1171, Promega Inc., Madison, Wisconsin, USA). Medium was removed 24 hours after transfection and replaced with 100 μl/well serum-free, substrate equilibration medium composed of CO_2_-Independent Medium (18045–054, Gibco, Life Technologies) containing 2 mM glutamine and 2% (v/v) 50 x Glo-Sensor^TM^ cAMP Reagent (30.6 mg/ml in10 mM HEPES (pH 7.5), Promega Inc.). Cells were incubated in equilibration medium for 1.5 h at 37°C and 5% CO_2_ to allow intracellular up-take of luciferase substrate. Prior to the actual measurement of ligand-induced cAMP signaling, baseline equilibration was performed. To this end luciferase activity was measured at 40 s intervals for 0.1 s each in a TriStar LB 941 luminescence reader (Berthold Technologies, Switzerland), using MikroWin 2000 software. Once a plateau was reached, synthetic MSH ligands were added and measurements were immediately continued at 40 s intervals. The increase in the intracellular cAMP concentration in response to MCR activation by MSH ligand was monitored as an increase in luminescence over time. To establish dose response curves, MCR and Glo-sensor transfected cells were incubated with serial dilutions of β-MSH or γ-MSH ligand ranging from 1 pM to 200 μM. Luminescence was also measured within the same plate in cells transfected with *IRES GFP* and Glo-sensor, to account for non-MCR mediated cAMP signaling through endogenous G-protein coupled cell surface receptors. The maximal luminescence reached after ligand addition was identified in each well of MCR- and Glo-sensor transfected cells and was subtracted by the value of baseline signaling measured in the plateau phase immediately before ligand addition. Furthermore, the increment of baseline corrected non-MCR signaling was determined for the same time point in wells with IRES GFP and Glo-sensor transfected cells, and was additionally subtracted. These double corrected relative luminescence values (RLU) thus reflect the maximal luminescence response induced by MCR-specific ligand binding at a given ligand concentration. To allow comparison between plates, RLU measurements for γ- and β-MSH peptides were always normalized plate internally to the RLU obtained with γ2-MSH and β-MSH H9, respectively, at their highest concentration (RLU of 1). Normalized RLUs were determined in triplicate on 3 different plates for each ligand and concentration and dose response curves calculated by non-linear regression curve fit (least square fit). In figure representations of dose response curves mean RLU and their standard errors of the mean (SEM) are plotted against LOG_10_ of the ligand concentration. In this way, ligand specific differences in the relative Emax. can be appreciated.

For determination of EC50, RLU were normalized in a way that the smallest and highest values in each ligand-specific data set were set to 0% and 100%, respectively, irrespective of the actual maximal signaling amplitude. The percentage of RLU was then plotted against LOG_10_ of the ligand concentration, and EC50 calculated from normalized dose response curves established by non-linear regression curve fit (least square fit).

To evaluate the relative signaling strength of various γ-MSH forms on MC3R, EC50 were calculated for each plate separately from normalized dose response curves, the inverse EC50 (1/EC50) was calculated for each ligand and normalized to the inverse EC50 of γ2-MSH. Statistical differences in the signaling strength were determined by unpaired t-test (n = 3, *p < 0.05, **p<0.01, ***p<0.001, ****p<0.0001).

### Measurement of the signaling activity of cloned *Tyto alba alba* (TA) *POMC* N-terminal cleavage site 2 and 3 fragments

HEK293T cells were transfected with plasmids coding for TA *POMC* N-terminal cleavage site 3 fragments with 3, 5, 7, 10 or 18 serine residues in the γ3-MSH locus, in combination with mouse *7B2* plasmid (285 ng of each of the two plasmids per 1.9 cm^2^ well). Transfections were performed in triplicate with Lipofectamine 3000. Supernatants (500 μl per well) were harvested 71 h post transfection, and frozen at -80°C. Secretion of N-terminal POMC fragments into the supernatant was confirmed in Western blot with anti-HA antibody. Bands were quantified densitometrically using ImageJ software (version 1.45s). Bands from N-terminal cleavage site 2 fragment and from all cleavage site 3 fragments, including the major glycosylated and minor non-glycosylated forms, were simultaneously outlined and the density profile was plotted across lanes. Pixel numbers were determined underneath individual peaks corresponding to the different N-terminal POMC fragment types. The resulting pixel densities were normalized to the density of TA POMC cleavage site 2 fragment to determine the relative expression levels. Supernatants were then serially diluted in serum-containing medium. The cAMP signaling activity was assessed for each dilution on TA *MC3R* and Glo-sensor transfected HEK293T cells (1 ng *MC3R* plasmid and 100 ng Glo-Sensor plasmid/well in 96 well plates). Luminescence measurements were simultaneously performed for the different types of N-terminal POMC fragments from one transfection series (cleavage site 2 and cleavage site 3 fragments with 3, 5, 7, 10 and 18 S), and transfection series 2 and 3 were analyzed in separate plates. Baseline signaling was subtracted from maximal, N-terminal POMC fragment-induced luminescence. Supernatant from mock-transfected cells (*IRES GFP* plasmid) served as negative control to exclude unspecific MC3R signaling by serum components or secreted cellular factors. The corrected maximal luminescence values were normalized in each transfection series to the response obtained for TA POMC N-terminal cleavage site 2 fragment at its highest concentration. Furthermore, the relative concentrations of N-terminal POMC fragments in supernatants were corrected for their relative expression levels determined by Western blot. The normalized, relative cAMP signaling responses of TA POMC N-terminal cleavage site 2 and 3 fragments with 3, 5, 7, 10 or 18 serine residues were then plotted against LOG_10_ of the corrected relative concentrations. Relative EC50 for all POMC N-terminal fragments were determined in triplicate from dose response curves of the three transfection series. The inverse relative EC50, reflecting the relative signaling activity, was calculated and normalized to the inverse relative EC50 of TA POMC cleavage site 2 fragment. Differences in the signaling activity of TA POMC N-terminal cleavage site 2 and 3 fragments were analyzed by one way ANOVA followed by Tukey’s multiple comparison test (n = 3, *p<0.05, **p<0.01, ***p<0.001, ****p<0.0001).

### Analysis of plasma and cerebrospinal fluid stability of different γ-MSH forms

For studies on the plasma stability of TA/SA γ-MSH, lyophilized citrated plasma from a pool of human donors was reconstituted in H_2_O as indicated by the manufacturer (# P9523, Sigma-Aldrich Chemie GmbH). Stability of human γ-MSH peptides was evaluated in fresh frozen human plasma (Interregional Blood Transfusion Swiss Red Cross Ltd, Epalinges, Swizerland). To this end, pooled samples of complete EDTA blood were centrifuged for 10 min at 1300 g, plasma was separated from buffy coat and erythrocytes and cleared by an additional 10 min centrifugation step at 1300 g. Cerebrospinal fluid pooled from ≥ 3 human donors was provided in liquid phase (# 991-19-P, Lee Biosolutions Inc., Maryland Heights, MO, USA). Aliquots of both body fluids were stored at -80°C until use. Samples of reconstituted plasma or CSF were thawed on ice, the pH of fresh frozen plasma was adjusted to 7.4 by addition of triethanolamine to a final concentration of 50 mM, and 45 μl aliquots were distributed to 1.5 ml Protein LoBind Tubes (Eppendorf AG, Hamburg, Germany). The γ-MSH synthetic peptides were pre-diluted in H_2_O to 20 μM, and 5 μl each were added on ice to 45 μl pre-laid samples of plasma or CSF. The γ-MSH starting concentration in degradation assays was thus 2 μM. Immediately after mixing, a first 5 μl aliquot for time point zero was removed, tubes were then transferred to a 37°C heat block, and further 5 μl aliquots were collected from plasma and CSF samples after various incubation times up to 68 h and 72.5 h, respectively. For control purposes, γ-MSH peptides were also diluted in H_2_O to a concentration of 2 μM and aliquots were removed after the corresponding incubation times at 37°C. All collected 5 μl aliquots were immediately diluted in 45 μl H_2_O and frozen on dry ice in 96 well V-bottom non-binding microplates (Greiner Bio-One Vacuette Schweiz GmbH, St. Gallen, Schweiz) to stop further degradation. After completion of degradation time courses, collection plates were stored at -80°C.

The residual TA/SA or human γ-MSH signaling activities were simultaneously analyzed on Glo-sensor and TA or human *MC3R* transfected HEK293T cells, respectively. 11 μl of diluted, γ-MSH-containing plasma or CSF samples were transferred to 100 μl substrate equilibration medium on transfected HEK293T cells in 96 well plates. Thus, for time point zero the final concentration of γ-MSH ligand on MC3R and Glo-Sensor expressing cells was 20 nM. Maximal luminescence values for the residual γ-MSH ligand activities were determined for all time points in the degradation time course, were baseline subtracted and normalized to the maximal luminescence value at time point zero for the same ligand in the same plate. All degradation time courses were performed in triplicate and read on three separate 96 well plates. Mean values of the relative residual signaling activities (RLU) were plotted against time. Degradation curves were established by non-linear regression curve fit (least square fit) and half-lives determined from one phase decay curves. Baseline signaling of plasma or CSF without any γ-MSH-ligand was likewise followed over time, and completeness of degradation of the γ-MSH ligand was thus reached when the signaling activity had dropped to the level of plasma or CSF signaling. We further verified that signaling of γ-MSH and its breakdown products was MC3R-specific and did not elicit signaling through any other endogenous, G-protein coupled cell surface receptors. To this end plasma and CSF digested γ-MSH was tested on IRES GFP and Glo-sensor expressing cells.

### Competition of *Tyto alba alba* (TA) β-MSH P9 with β-MSH H9, γ2-MSH or γ3-MSH in MC3R and MC4R signaling

To address the question whether TA β-MSH P9 is dominant or recessive, we analyzed signaling of the TA β-MSH H9 variant at 20 nM on MC3R, and 50 nM on MC4R in the presence of increasing concentrations of Tyto alba β-MSH P9 (0 to 5 μM). Baseline corrected maximal luminescence values were normalized to β-MSH H9 signaling at 50 nM and 20 nM on MC3R and MC4R, respectively, in the absence of β-MSH P9.

To exclude that β-MSH P9 acts as a competitive antagonist to γ2- or γ3-MSH, the cAMP signaling responses of 10 nM TA γ2-MSH or 10 nM TA γ3-MSH 10 S were analyzed in the presence of increasing concentrations of TA β-MSH P9 (0 to 2 μM) on HEK293T cells transfected with TA *MC3R* or *MC4R* and *Glo-Sensor* (see above). Signaling of TA β-MSH P9 alone was analyzed in parallel. Maximal luminescence values in the γ2-MSH + β-MSH P9 series, and the γ3-MSH 10 S *+*β-MSH P9 series were identified, baseline subtracted, and normalized to the baseline corrected signaling response of 10 nM γ2-MSH or γ3-MSH 10 S in the absence of β-MSH P9, respectively. The TA β-MSH P9 dose response curve in the absence of any γ-MSH was normalized to signaling of 10 nM γ2-MSH within the same plate.

## Results

### A H9P mutation in β-MSH is linked to a polymorphic poly-serine stretch in γ3-MSH in *POMC* of Tytonidae

**Fig 1:**
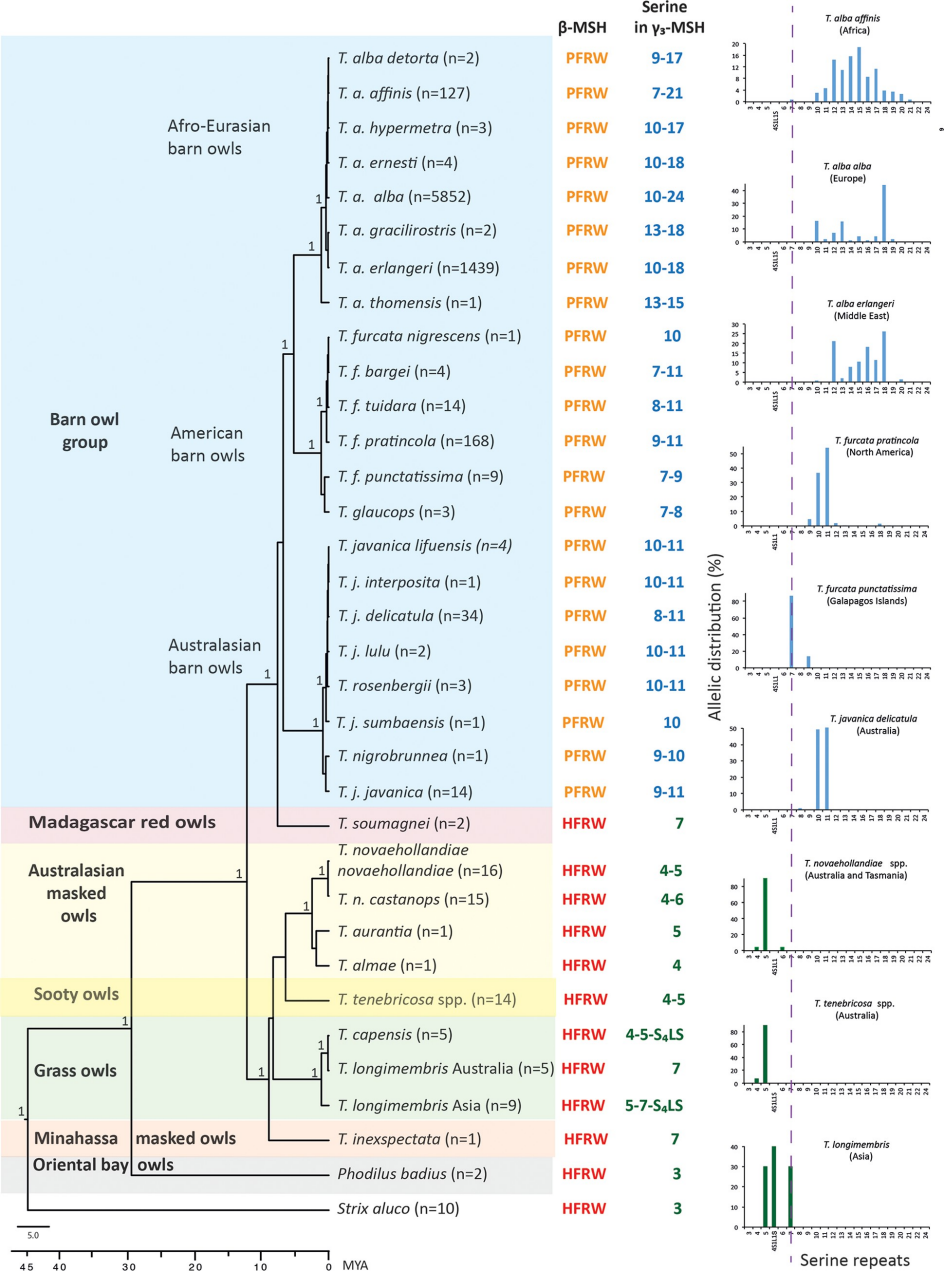
Molecular phylogeny of the Tytonidae family, polymorphism in β-MSH at the receptor-binding site (ancestral HFRW or mutated PFRW), and number of serine repeats in γ3-MSH. Tytonidae taxa homozygous for HFRW are written in red and taxa carrying the homozygous mutation PFRW are written in orange. The range of the number of serine repeats is indicated in blue when ≥ 7 and in green when ≤ 7 and N indicates the number of sequenced individuals. For taxa with a large number of individuals sequenced, we report the distribution of the number of serine repeats. In the phylogenetic tree, 1 indicates that the node has a posterior probability of 1 (= 100%) and the time unit is million years (MYA).

Genetic screening of wild-living barn owl species uncovered a close association between the mutation H9P in β-MSH and an unusual poly-serine extension in the γ3-MSH locus of *POMC*. A preliminary comparative analysis of *POMC* sequences across Strigiformes (the order including Tytonidae and its sister group Strigidae) showed that owl species from the Strigidae family carry 3 serine residues in the homologous γ3-MSH position and β-MSH H9 wild-type alleles. This includes the tawny owl *Strix aluco* (SA), used as a comparative outgroup to Tytonidae in this study (Fig 1). These observations suggested that the polymorphisms in the γ3- and β-MSH loci found in Swiss barn owls may be restricted to Tytonidae, which prompted us to investigate the evolution of the *POMC* sequence throughout the phylogeny of this family.

The Tytonidae originated about 45 million years ago (MYA) and contain only two genera (*Tyto* and *Phodilus*) that split 28 MYA (Fig 1). *Tyto alba*, the Afro-Eurasian (or Western) barn owl, is part of the barn owl group, which additionally includes two sister lineages, the Australasian (or Eastern) barn owl *Tyto javanica* and the American barn owl *Tyto furcata* (Fig 1). The barn owl group is sister to the rare Madagascar red owl, *Tyto soumagnei*, with whom it may have shared a common ancestor *ca*. 8 MYA (Fig 1). The remaining *Tyto* species, grass-, sooty- and masked owls belong to a distinct clade that may have shared a common ancestor with the barn owl group and the Madagascar red owl *ca*. 12 MYA (Fig 1).

Similar to SA and other Strigidae, *Phodilus* and most *Tyto* species bear the wild-type H9 at the **H**FRW receptor-binding motif of β-MSH (Fig 1). The homozygous proline mutation in position 9 (P9) that alters the receptor-binding site of β-MSH from **H**FRW to **P**FRW was exclusively present in the barn owl group, including the European *Tyto alba alba* (TA) (Fig 1). The inspected Madagascar red owls, sister to the barn owl group, were found homozygous for the β-MSH H9 allele, suggesting that the P9 mutation might have appeared after the split between these two groups and before the barn owl group diverged into different lineages (*ca*. 6 MYA).

In the barn owl group, the **P**FRW motif in β-MSH co-presents with poly-serine stretches ranging from 7 to 24 repeats in the γ3-MSH locus. In contrast, *Tyto* species bearing **H**FRW in β-MSH contain 4 to 7 serine repeats in γ3-MSH, and *Phodilus badius* and the outgroup tawny owl *Strix aluco* (SA) present with only 3 serine repeats. Thus, while the poly-serine stretches extended >3 repeats in all *Tyto* species, they show substantial extension specifically within the barn owl group, in conjunction with the H9P mutation in the β-MSH locus (Fig 1). The genetic link between the H9P mutation in β-MSH and serine extension to ≥ 7 repeats in γ3-MSH, unique to the barn owl group, may therefore have evolved only once in the common ancestor of this group.

**Fig 2:**
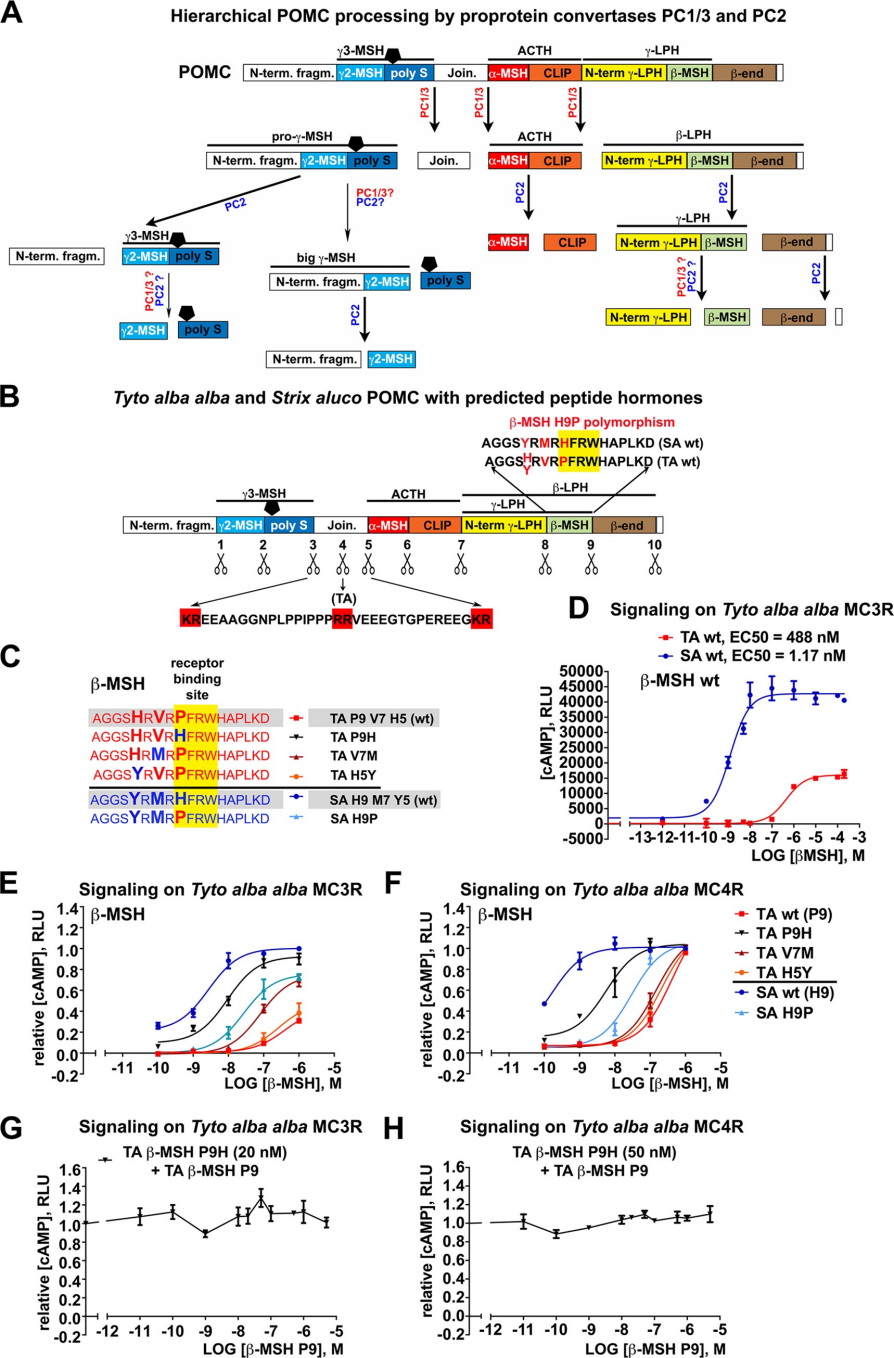
The H9P mutation in *Tyto alba alba* β-MSH abrogates receptor signaling. (A) Summary on the hierarchical processing of POMC by proprotein convertases PC1/3 and PC2 [4, 31–33]. (B) Schematic representation of *Tyto alba alba* (TA) / *Strix aluco* (SA) POMC with putative dibasic cleavage sites indicated by scissors. Note the additional RR motif within the Joining peptide in TA. The amino acid (aa) sequences of β-MSH for both owl species are indicated above the polypeptide precursor. (C) Synthetic β-MSH peptide variants used to assess receptor signaling. The naturally occurring β-MSH variants in SA and TA are under-laid in gray and are designated as wild-type (wt), with differing aa depicted by enlarged letters. In artificial variants, aa in agreement with TA or SA wt forms are in red and blue, respectively. Wt TA β-MSH variants carry proline in position 9 (P9) as part of the **P**FRW receptor-binding motif (yellow), whereas β-MSH of SA displays histidine in position 9 (H9). (D-F) Receptor-signaling of β-MSH variants. Recombinant TA MC3R or MC4R were transiently expressed in HEK293T cells, followed by exposure to synthetic β-MSH peptides at the indicated concentrations. Receptor signaling activity was assessed by monitoring cellular cAMP levels via Glo-sensor in a luminescence assay as detailed in Materials and Methods. Data are means + SEM, n = 3. Dose-response curves were calculated by non-linear regression analysis. (G, H) TA β-MSH P9 cannot compete with the hypothetic TA β-MSH H9 form in receptor signaling, neither on MC3R (G) nor MC4R (H).

Considering that β-MSH and γ3-MSH are both derived from the POMC precursor through cleavage by PC1/3 and PC2 (Fig 2A) and bind the same receptors, we hypothesized a functional link between the two polymorphisms. First, we tested the effect of the H9P mutation on the receptor signaling capacity of β-MSH. We then investigated the impact of the poly-serine polymorphism in the γ3-MSH locus on the processing of the POMC precursor, the receptor signaling properties of the γ3-MSH peptide, and the plasma stability of γ3-MSH.

### The H9P mutation in *Tyto alba alba* (TA) β-MSH abrogates receptor signaling

In mammals, β-MSH exerts its major physiological functions by signaling through MC3R and MC4R involving the second messenger cAMP [3, 29]. To test whether the H9P mutation found in the barn owl group affects the receptor signaling activity of β-MSH, we compared TA β-MSH P9 with β-MSH H9 from SA (Fig 2B and 2D). Sequence comparison revealed significant differences between owl-derived MC3R and MC4R and available orthologues from other species. We therefore generated recombinant forms of TA MC3R and MC4R. To quantitatively assess MCR activation by β-MSH variants, we employed a robust cell-based assay for detection of intracellular cAMP levels [30]. Briefly, human HEK293T cells were transiently co-transfected with TA MC3R or MC4R in combination with a plasmid expressing GloSensorTM-20F cAMP that allows sensitive and reliable quantification of intracellular cAMP levels via a luciferase reporter, as detailed in Materials and Methods. At 26 h post transfection, cells were incubated with increasing concentrations of TA β-MSH P9 and SA β-MSH H9, followed by detection of cellular cAMP levels by GloSensor assay. Dose-response characteristics revealed >400-fold increase in EC50 on TA MC3R with TA β-MSH P9 compared to β-MSH H9 of SA (Fig 2D). The EC50 of SA β-MSH H9 was comparable to the EC50 observed for human β-MSH H9 on the human MC3R orthologue in our system, further validating our system (Fig 6F–6I). The maximal signaling amplitude (E_max_) of TA β-MSH P9 was likewise reduced, indicating a marked decrease in MC3R signaling of TA β-MSH P9 compared to SA β-MSH H9 (Fig 2D).

To validate the specific contribution of the H9P mutation to the impaired signaling activity of TA β-MSH, we introduced the reverse P9H mutation into TA β-MSH (TA P9H) and H9P into SA β-MSH (SA H9P) (Fig 2C). The H9P mutation reduced the TA MC4R and MC3R signaling of SA β-MSH by >100-fold and 10-fold, respectively (Fig 2E and 2F). The reciprocal P9H mutation enhanced TA MC4R and MC3R signaling of TA β-MSH by almost 100-fold. In addition to P9 / H9, TA and SA β-MSH differ in two residues, V7 / M7 and H5 / Y5, upstream of the receptor-binding site (Fig 2B and 2C). The substitutions V7M and H5Y had no significant effect on TA MC4R signaling of TA β-MSH (Fig 2F) and only mildly affected TA MC3R signaling (Fig 2E), pinpointing P9 as the crucial mutation linked to the functional impairment of TA β-MSH. It is conceivable that the marked reduction in MCR signaling activity of β-MSH P9 may be due to loss of receptor binding. To address this point, we performed a competition assay measuring receptor-signaling activity of an artificial, functionally active TA β-MSH H9 variant in presence of an increasing molar excess of defective TA β-MSH P9. As shown in Fig 2G and 2H, a molar excess of β-MSH P9 hardly affected β-MSH H9 receptor signaling, suggesting that β-MSH P9 is unable to compete with β-MSH H9 for receptor-binding. In sum, the data indicate that the H9P mutation in *Tyto alba alba* β-MSH abrogates receptor signaling.

### The poly-serine stretch in the γ3-MSH locus affects PC cleavage of POMC

**Fig 3:**
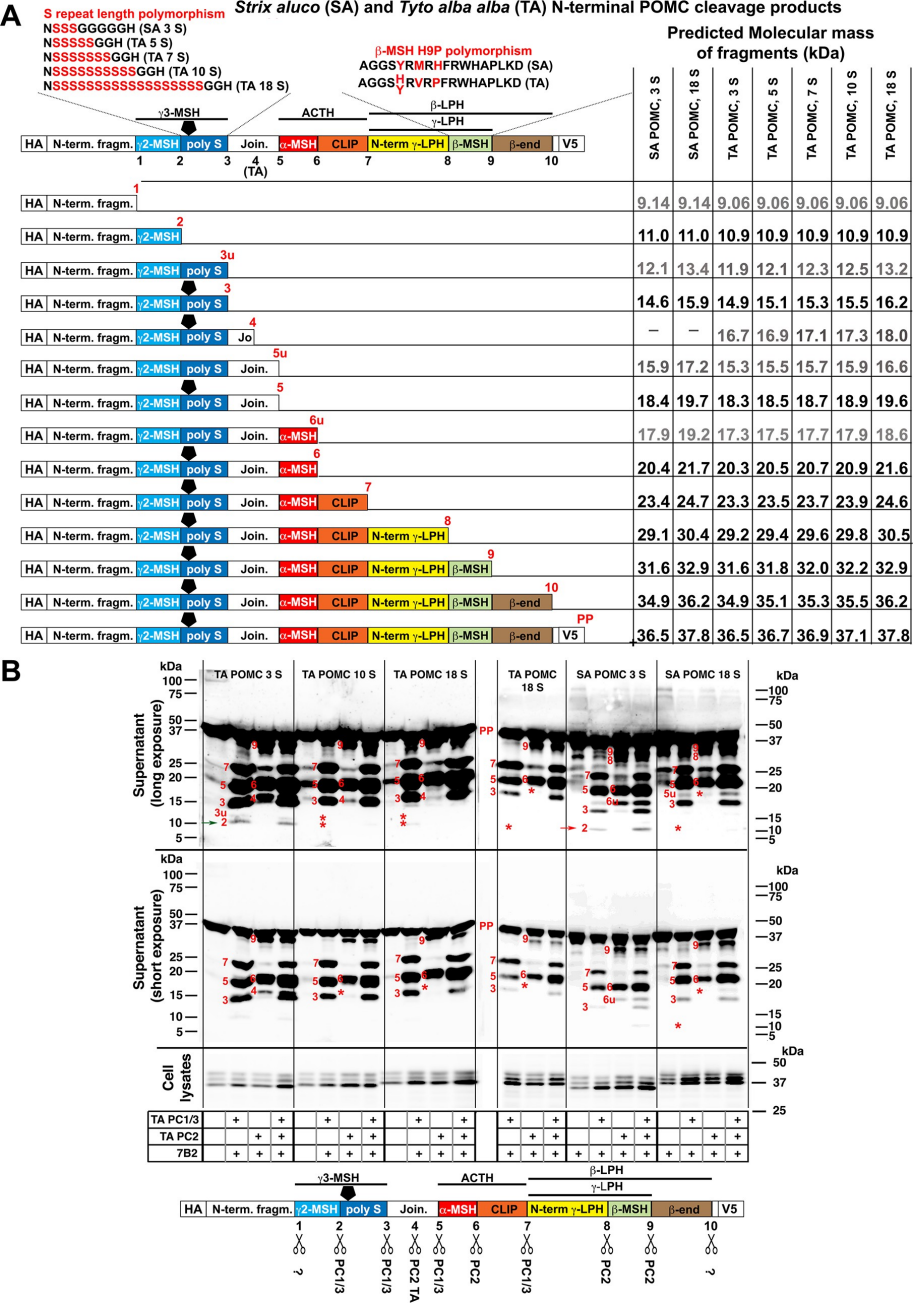
The poly-serine stretch in the γ3-MSH locus of POMC affects PC cleavage. (A) Schematic representation of recombinant double-tagged (HA, V5) SA / TA POMC precursor with dibasic cleavage sites 1 to 10, required for production of the peptide hormones γ2-MSH, γ3-MSH, ACTH, α-MSH, CLIP, β-LPH, γ-LPH, β-MSH, and β-end. The N-glycan at the asparagine residue within γ3-MSH is depicted as black pentagon. The polymorphisms in γ3-MSH and β-MSH are shown above the POMC precursor. N-terminal cleavage fragments up to sites 1 through 10 are listed below the precursor, and corresponding bands in the below Western blots are labeled by the according numbers in red. Fragments 3u, 5u, 6u likely correspond to under-glycosylated forms of fragments 3, 5 and 6, respectively. The full length POMC precursor is labeled with PP. The indicated molecular masses of fragments were calculated as detailed in Supplemental Information and S4 Fig. (B) Cleavage of TA / SA POMC variants by TA PC1/3 and PC2 (with 7B2) in HEK293T cells. Cell lysates and supernatants were harvested 56 h post transfection and N-terminal POMC fragments were detected in Western blot with an anti-HA antibody. Note that the PC1/3-specific fragment resulting from cleavage at site 2 (red arrow) is present in wt SA POMC with 3 serine residues, but not in TA POMC with 10 or 18 serine residues (asterisks indicating missing fragments produced by the respective PC). Reducing the number of serine residues in TA POMC to 3 leads to re-appearance of band 2 (green arrow), and increasing the number of serine residues in SA POMC to 18 to its disappearance. Identified PC1/3 and PC2 specific cleavage sites in SA and TA POMC are indicated in the bottom schematic below the scissors.

The proximity of the poly-serine polymorphism in γ3-MSH to PC cleavage sites 2 and 3 within the POMC precursor opened the possibility that the number of flanking serine repeats may influence the efficiency of PC processing (Fig 3A). Although PC substrate selectivity is mainly defined by the residues at the dibasic recognition site K/R(X)_n_K/R↓, flanking regions can affect accessibility to the consensus site [34]. While the tissue-specific expression of *POMC*, *PC1/3*, and *PC2* in humans and rodents is well established, no information was available for barn owls. Since barn owls are a protected species, we examined expression of *POMC*, *PC1/3*, and *PC2* mRNA in a specimen of TA that had died of natural causes. Relatively high levels of *POMC*, *PC1/3*, and *PC2* mRNAs were detected in the brain in cortex, hypothalamus, and pituitary, confirming the presence of both relevant PCs in tissues linked to endocrine and paracrine production of POMC and its derived peptide hormones (S2 Fig).

To investigate possible effects of the poly-serine polymorphism on processing of the owl-derived POMC, we cloned TA orthologues of PC1/3 and PC2. Recombinant TA PC1/3 and PC2 showed comparable expression in HEK293T cells and proper secretion was confirmed in supernatants (S3A Fig). Enzymatic activity was examined employing a well-established *in vitro* enzymatic assay using the canonical basic PC fluorogenic peptide Pyr-ERTKR-AMC as substrate, as detailed in Materials and Methods. The barn owl-derived PC1/3 and PC2 were maximally active at mildly acidic pH in line with their known activity in acidified secretory granules [35] (S3B Fig). As expected, both owl-derived PCs exhibited calcium-dependence (S3C Fig), a hallmark of their mammalian orthologues. Activity of TA PC2, but not PC1/3, critically depended on the co-expression of the chaperone 7B2, which is known to be required for proper folding and transport of mammalian PC2 [36, 37] (S3D Fig). Interestingly, endogenous basic PCs in HEK293T cells, e.g. PC2 or furin [38], did not yield significant cleavage of the fluorogenic peptide Pyr-ERTKR-AMC after shedding into the supernatant, as shown in mock transfected cells (S3B Fig). In sum, the data at hand indicated that the PC1/3 and PC2 variants obtained from TA were fully active and shared key characteristics of their mammalian counterparts.

Next, we defined the yet unknown TA PC1/3- and PC2-specific cleavage pattern of TA and SA POMC. The sequence of SA POMC contains nine putative basic PC cleavage motifs, similar to human POMC, whereas TA POMC contains an additional dibasic site within the joining peptide (Fig 2B), resulting in ten putative cleavage sites. In order to label corresponding cleavage sites in both owl species consistently, we shifted SA POMC cleavage sites downstream of the joining peptide by +1 (Fig 3A). The reported hierarchy of mammalian POMC processing with initial cleavage at the ACTH-flanking sites 5 and 7 renders detection of site 2 and 3 cleavage products *via* the C-terminal V5 tag difficult. The POMC cleavage fragments were therefore visualized by Western blot using the N-terminal influenza hemagglutinin (HA)-tag, which allowed sufficient resolution. To define the identity of POMC cleavage products, we generated a “ladder” comprised of a panel of SA POMC fragments reaching from the N-terminus to cleavage sites 1, 2, and 3 (S4A Fig). The individual N-terminal SA POMC fragments served as markers to identify the specific cleavage products generated by processing of full-length SA POMC with PC1/3 and PC2 (S1 Data and S4 Fig).

After calibration of our system, we compared the PC-specific cleavage pattern in SA and TA POMC. To this end, HEK293T cells were transfected with SA or TA POMC bearing varying numbers of serine residues, together with plasmids coding for TA PC1/3 and/or PC2 and 7B2. Supernatants were harvested and analyzed for cleavage products in Western blot (Fig 3). Comparable expression of POMC precursor, PC1/3 and PC2 was verified in cell lysates (Fig 3B, S5A Fig and S5B Fig). POMC carries a single N-glycosylation motif NXS/T [39, 40] within the γ3-MSH coding sequence. The three bands detected for POMC precursor in lysates were identified as the mature complex N-glycosylated from, the high-mannose intermediary glycosylation product, and the unglycosylated POMC precursor (Fig 3B and S1 Fig). In the supernatant, we detected predominantly the mature N-glycosylated form (S6B Fig). Co-expression with PC1/3 or PC2 yielded the expected complementary cleavage patterns for POMC in both SA and TA (Fig 3B). As in humans, cleavage of the owl-derived POMC precursors by PC1/3 yielded ACTH (Fig 3B), suggesting conserved PC specificities across species. PC1/3 was required for the production of γ3-MSH and γ2-MSH (Fig 3B). The expected product of cleavage at site 1 representing the N-terminal fragment of POMC was not detected, suggesting either inefficient processing or rapid degradation. Human POMC contains an O-glycosylation site at position T45 [41], which may contribute to stability of the N-terminal fragment. Owl-derived POMC bears S in the homologous position, but its O-glycosylation state is unclear.

The poly-serine polymorphism at the γ3-MSH locus of TA affected processing at the flanking cleavage sites 2 and 3, required for the production of γ2-MSH and γ3-MSH, respectively. Specifically, the N-terminal fragment resulting from site 2 processing by PC1/3 was detected in PC1/3-digested SA POMC with 3 serine residues, but seemed absent from TA POMC containing 10 or 18 serine residues (Fig 3B). Artificially increasing the number of serine residues from 3 to 18 in SA POMC resulted in a similar apparent loss of site 2 cleavage by PC1/3. Reciprocally, shortening the poly-serine stretch in TA from 18 to 3 enhanced site 2 processing. The modulation of PC1/3-mediated site 2 processing by variation of the number of the flanking serine residues in γ3-MSH of both owl species provided first evidence that the poly-serine polymorphism could influence POMC cleavage.

### Expansion of the poly-serine stretch increases the γ3-MSH/γ2-MSH ratio

**Fig 4:**
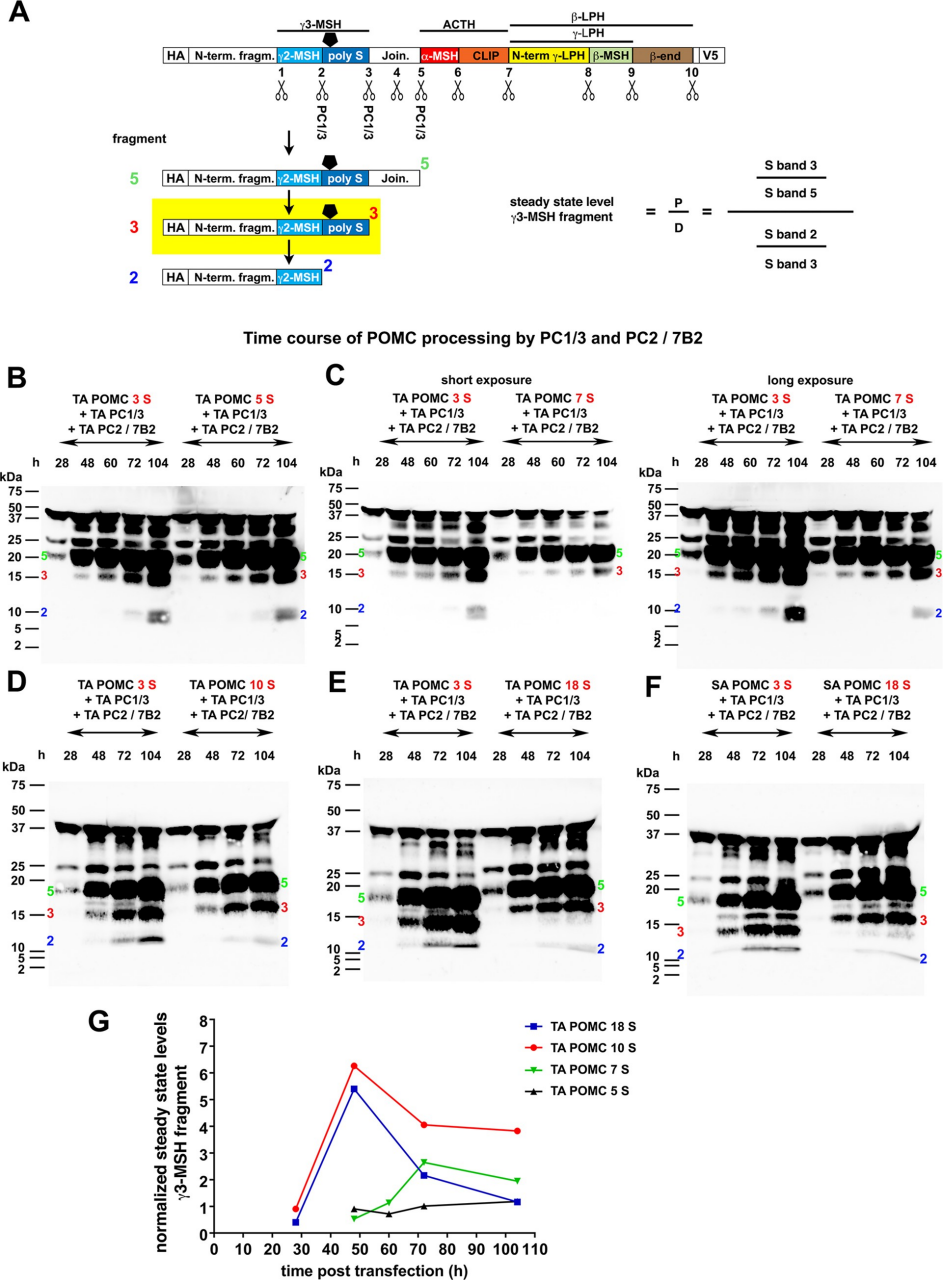
The poly-serine stretch in the γ3-MSH locus of POMC affects γ3-MSH steady-state levels. (A) Schematic figure explaining the assessment of steady-state levels of TA γ3-MSH fragment (under-laid in yellow). The immediate fragment 5 precursor is cleaved from full length POMC (top, as in 3B) and is further trimmed to fragments 3 and 2, whereby all cleavage steps are mediated by PC1/3. Steady-state levels of TA γ3-MSH fragment are operationally defined as the ratio of its production rate (P) / degradation rate (D). The production rate is calculated from the ratio of the signals S_band3_ / S_band5_ and the degradation rate from the ratio S_band2_ / S_band3_ as indicated in the formula. (B-F) HEK293T cells were transfected with TA *POMC* containing 3, 5, 7, 10 or 18 serine residues in the γ3-MSH locus or SA *POMC* containing 3 or 18 serine residues together with *PC1/3*, *PC2* and *mouse 7B2*, as in 3B. Aliquots of supernatant were harvested at the indicated time points, and N-terminal POMC cleavage products detected in Western blot with an anti-HA antibody. Bands corresponding to the N-terminal cleavage site 5, 3 and 2 fragments, labeled in green, red and blue, respectively, were quantified by densitometry. (G) Quantification of the steady-state levels of γ3-MSH fragment over time based on blots B-F. For each POMC species and time point indicated, the steady state levels of γ3-MSH fragment were determined as outlined in A, using the same blots with suitable exposure times, and normalized to the steady sate level of artificial TA γ3-MSH fragment with 3 serine residues at the corresponding time point within the same blot (internal standard).

Our phylogenetic examination revealed that the presence of the H9P loss-of-function mutation in β-MSH was associated with a sharp transition in the length of the poly-serine stretch to ≥7 residues (Fig 1). We therefore examined the time course of PC1/3 and PC2 cleavage of TA POMC containing increasing numbers of serine residues in the γ3-MSH locus (Fig 4). SA POMC variants containing 3 or 18 serine residues in the γ3-MSH locus served as benchmarks. TA POMC with 3 serines and SA POMC containing 18 serines do not naturally occur and were included to assess the influence of the poly-serine stretch across species. Recombinant TA POMC variants with 3, 5, 7, 10 or 18 serine repeats were co-expressed with TA PC1/3 and PC2, and supernatants sampled at the indicated time points. Comparable levels of POMC precursor and PC expression were verified by Western blot in cell lysates (S6A Fig and S6B Fig). Examination of PC processing over time revealed that TA POMC containing 5 serine residues underwent cleavage with kinetics comparable to the TA construct with 3 serine repeats (Fig 4B), whereas variants containing ≥7 residues showed markedly delayed processing at both sites 2 and 3 (Fig 4C–4E). As expected, extension of the poly-serine stretch from 3 to 18 in the γ3-MSH locus of SA POMC delayed site 2 cleavage (Fig 4F) with kinetics comparable to the TA variant with 18 serine residues (Fig 4E).

The observed reduction in cleavage at sites 2 and 3 in POMC with ≥ 7 serine residues differentially affects γ3-MSH levels. Specifically, inhibition of site 3 cleavage decreases the production rate of γ3-MSH from the N-terminal fragment 5 precursor, whereas inhibition of site 2 cleavage increases γ3-MSH levels at the expense of γ2-MSH. To assess the net effect on γ3-MSH levels, we quantified the signals corresponding to N-terminal cleavage fragments 5, 3 and 2 and calculated production- and degradation rates of γ3-MSH (Fig 4A–4G). The resulting steady-state levels of γ3-MSH derived from individual POMC variants were then normalized for each time point to the corresponding artificial TA POMC 3 S variant that served as an internal standard. As shown in Fig 4G, extension from 3 to 5 serines hardly affected γ3-MSH steady-state levels. Extension to 7 serine residues resulted in a modest (2-3-fold) increase. In contrast, presence of poly-serine repeats of 10 or 18 residues lead to a robust (>5-fold) increase of γ3-MSH levels after 48 h (Fig 4G). Although semi-quantitative in nature, our analysis revealed that expansion of the poly-serine stretch increases the γ3-MSH / γ2-MSH ratio.

### Differential MC3R signaling by γ2-MSH and γ3-MSH

**Fig 5:**
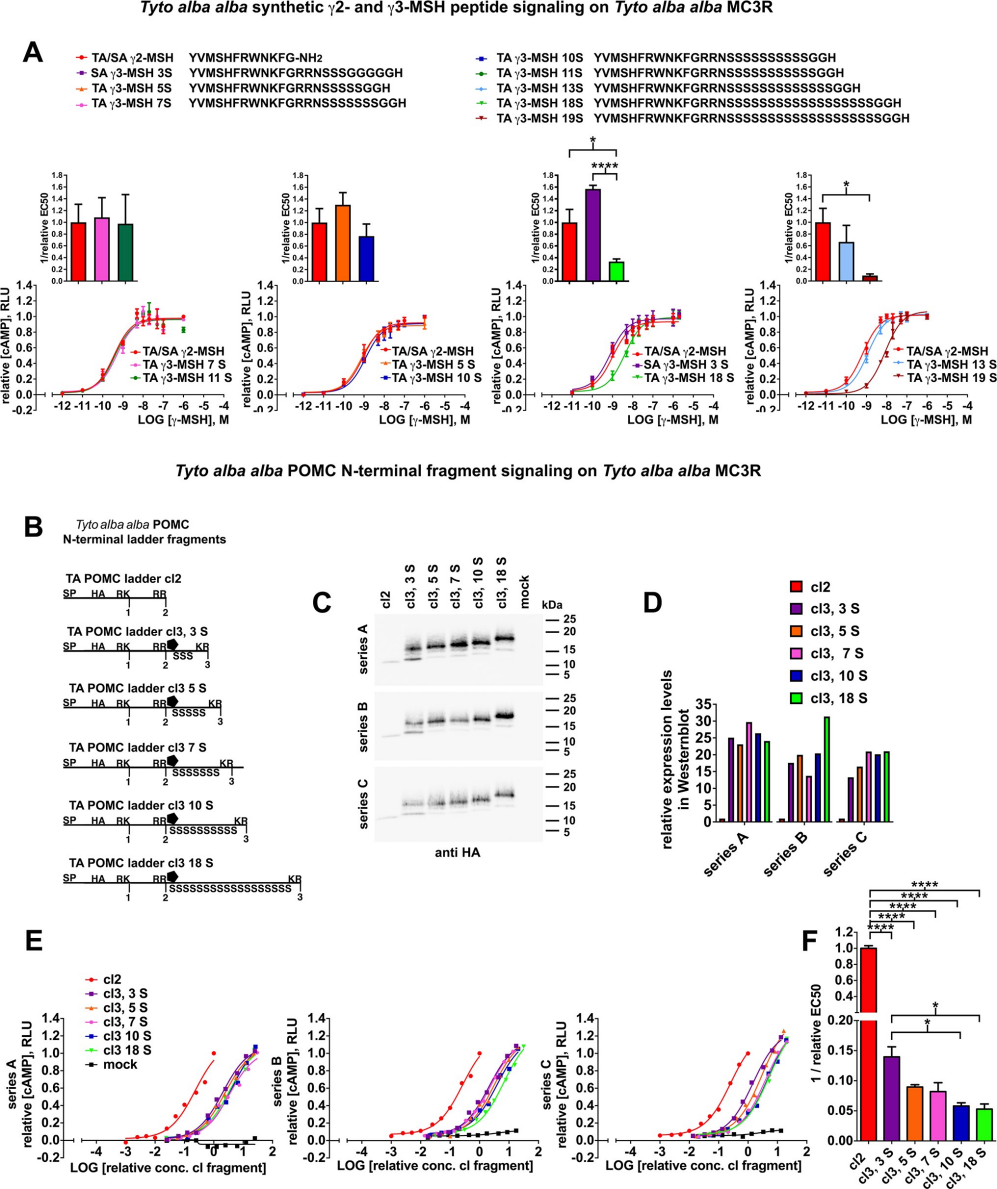
The signaling activity of N-glycosylated γ3-MSH on MC3R is reduced compared to γ2-MSH. (A) Receptor signaling of synthetic, non-glycosylated γ2- and γ3-MSH peptides (aa sequences depicted at the top). HEK293T cells were transiently transfected with expression plasmids for the TA *MC3R* and Glo-sensor. After 26 h, cells were exposed to the indicated synthetic peptides at the given concentrations. Dose-response curves were established by non-linear regression curve fit and normalized to the signaling activity of γ2-MSH at 1 μM. The relative signaling activities of γ3- compared to γ2**-**MSH were calculated from normalized dose response curves (highest value = 1, lowest value = 0) and expressed as 1 / relative EC50 (n = 3 dose response curves per peptide, statistical differences were determined by unpaired t-test, *p < 0.05, **p<0.01, ***p<0.001, ****p<0.0001). (B) Schematic representation of recombinant N-terminal TA POMC cleavage site 2 and 3 fragments including from N- to C-terminus: the POMC signal peptide (SP), the HA epitope-tag, the mature N-terminal POMC sequence up to cleavage site 2 (cl2) or cleavage site 3 (cl3), with 3, 5, 7, 10 or 18 serine residues in the γ3-MSH locus. (C) The constructs in B were transiently expressed in HEK293T cells and detected in supernatants by Western blot 71 h post transfection (triplicates). Please note the presence of characteristic upper bands corresponding to N-glycosylated forms (compare with S4 Fig). (D) Densitometric quantification of C. (E) Supernatants from transfected cells were serially diluted and signaling activity on TA MC3R assessed by Glo-sensor assay as in 5A. Relative concentrations were corrected for the expression levels in cell supernatants (C, D) and maximal luminescence values normalized in each transfection series to the maximal signaling activity of TA POMC cl2 fragment at its highest concentration. Dose-response curves were established (E). Supernatants from mock-transfected cells served as negative controls (mock). (F) Relative EC50 values for the different N-terminal TA POMC cl2 and cl3 fragments were calculated from dose response curves in E (n = 3), and the signaling activity (1 / relative EC50) was determined and normalized to 1 / relative EC50 of TA POMC cl2 fragment from the same transfection series. Mean values and SEM are depicted (n = 3). Differences were evaluated by ANOVA followed by Tukey’s multiple comparison test (*p<0.05, **p<0.01, ***p<0.001, ****p<0.0001).

Next, we characterized the receptor signaling properties of γ2-MSH and γ3-MSH variants. To this end, we used a panel of synthetic peptides representing γ2-MSH and the γ3-MSH variants listed in Fig 5A. Our quantitative receptor-signaling assay revealed only minor differences among γ2-MSH and γ3-MSH variants with up to 13 serine residues (Fig 5A). Only the γ3-MSH forms containing 18 and 19 serine residues showed reduced cAMP signaling on MC3R (Fig 5A).

As mentioned above, biosynthetic γ3-MSH bears a conserved N-glycan in P2’ position of cleavage site 2, which is absent from γ2-MSH. Since this modification was not present in the chemically synthesized peptides used in Fig 5A, we generated recombinant, fully glycosylated TA N-terminal POMC fragments ending at cleavage site 3 (pro-γ-MSH) and carrying 5, 7, 10 or 18 serine residues (Fig 5B). The non-natural *Tyto* pro-γ-MSH form with 3 serine residues as well as the non-N-glycosylated, N-terminal POMC fragment ending at cleavage site 2, corresponding to γ2-MSH were added as benchmarks. Expression of TA pro-γ-MSH in HEK293T cells resulted in correct N-glycosylation (S4F Fig). Upon transient expression in mammalian cells, conditioned supernatants were harvested, serially diluted, and TA MC3R signaling activity monitored. Data were normalized to the expression levels in supernatants determined by Western blot (Fig 5C and 5D) and relative signaling activities calculated (Fig 5E and 5F). All pro-γ-MSH fragments ending at cleavage site 3 and including the N-glycan showed >10-fold reduced signaling activities on MC3R when compared to the site 2 fragment corresponding to γ2-MSH. In summary, our data indicate reduced signaling activity of γ3-MSH on MC3R when compared to γ2-MSH, possibly due to steric hindrance by the N-glycan in position P2’.

### Serine repeats affect the plasma stability of γ3-MSH

**Fig 6:**
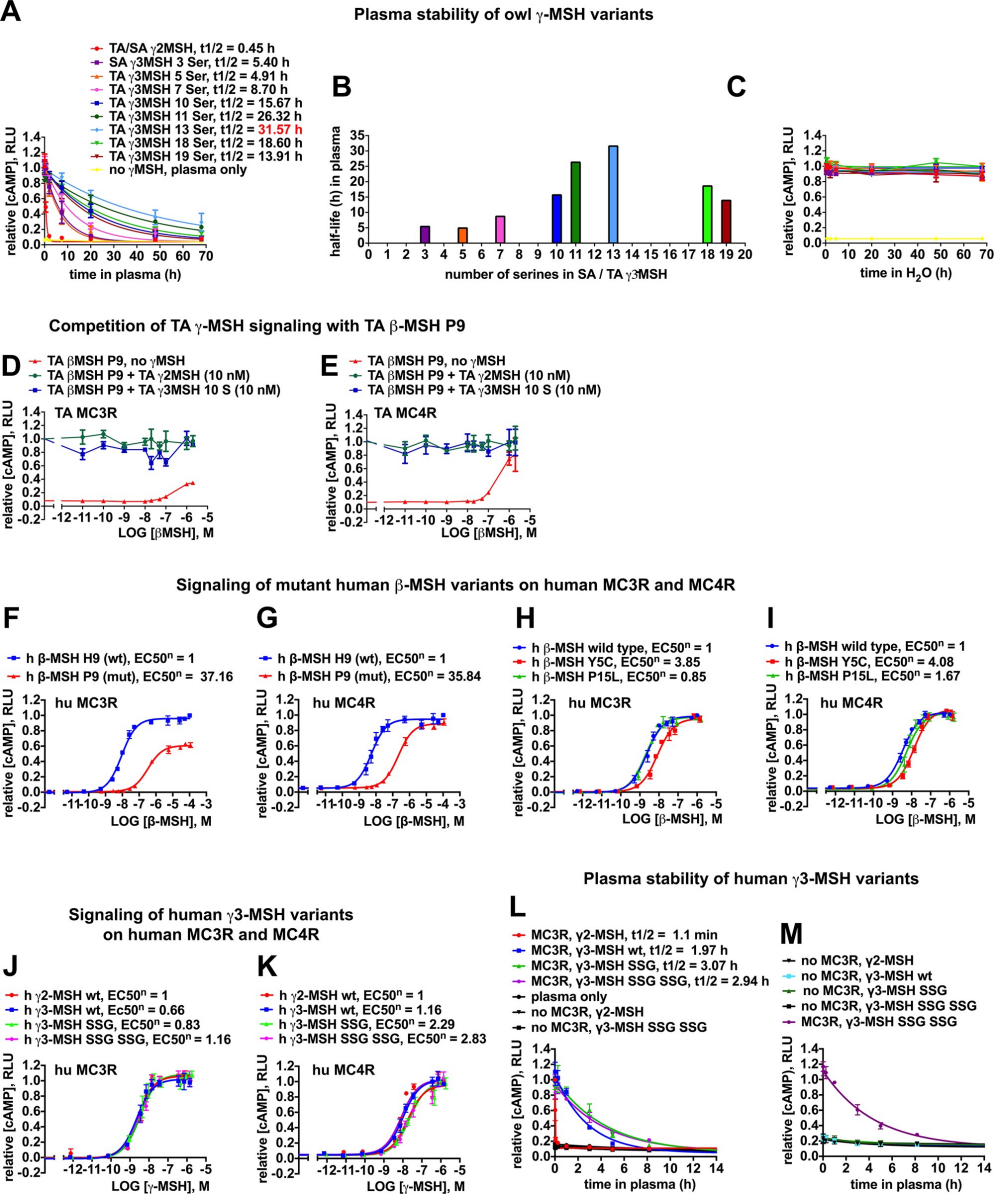
The β-MSH H9P loss of function mutation and increased plasma stability of γ3-MSH *vs*. γ2-MSH are conserved between owls and humans. Synthetic peptides of TA and SA γ2-MSH, SA γ3-MSH with 3 serine residues, or TA γ3-MSH with 5, 7, 10, 11, 13, 18 and 19 serine residues were diluted in human plasma (A), or water (C) to final concentrations of 2 μM and incubated at 37°C. Incubations for all γ-MSH peptides were performed in triplicate. Aliquots were removed after different time points for up to 68 h, and the remaining cAMP signaling activity was simultaneously determined on TA MC3R and Glo-sensor transfected HEK293T cells by luminescence assay. Measured maximal luminescence values were normalized to values at time point zero and half-lives were determined from one-phase decay curves (A, B). γ2-MSH, γ3-MSH and their breakdown products in blood plasma did not induce any signal on non-MC3R, but Glo-sensor transfected cells (S7A Fig). No loss in signaling activity was observed in water up to 68 h neither for γ2- nor for γ3-MSH, therefore excluding non-enzymatic decay or signaling loss over time due to peptide aggregation (C). (D, E) The inactive TA β-MSH P9 with its dysfunctional receptor-binding site PFRW does not act as a competitive antagonist for γ-MSH signaling on MC3R or MC4R, neither for γ2-MSH nor for γ3-MSH 10 serine variant. (F-I) Dose response curves for human MC3R (F, H) and MC4R signaling (G, I) of synthetic human β-MSH H9 (wild-type, Y5, P15), and mutant β-MSH P9, β-MSH Y5C and β-MSH P15L peptides. Data are means + SEM, n = 3. (J-K) Signaling of synthetic human non-glycosylated γ2- and γ3-MSH peptides (wild-type or with one or two SSG extensions in the serine-rich tail) on MC3R (J) and MC4R (K). Data are means + SEM, n = 3. EC50 were determined from dose response curves established after non-linear regression curve fit for the different human β- and γ-MSH peptides as described (Figs 2D–2F and 5A) and were normalized (EC50^n^) to the EC50 obtained for wild-type human β- (F-I) and γ2-MSH peptides (J, K). (L, M) Plasma stability of human γ2-MSH or γ3-MSH peptides (wild-type or γ3-MSH SSG or γ3-MSH SSG SSG) was assessed on human MC3R as described above for owl γ-MSH peptides on TA MC3R (A). Background signaling of residual human γ3-MSH peptides was established on non-MC3R, but Glo-sensor transfected cells (L, M).

So far, our studies uncovered that poly-serine extension to ≥7 residues in the γ-MSH locus of TA POMC increased the γ3-MSH/γ2-MSH ratio and that the signaling activity of γ3-MSH is reduced compared to γ2-MSH. Lastly, we investigated whether the length of the serine repeats affected the stability of γ3-MSH in plasma with possible relevance for its endocrine function. To this end, we employed a well-established *in vitro* assay to assess the plasma stability of peptide hormones, as detailed in Materials and Methods. Since both TA and SA are protected species, plasma sampling in sufficiently high amounts was not feasible. Based on our previous experience, lack of stringent quality control made chicken plasma obtained from slaughterhouses not suitable for our purposes neither. Considering the overall sequence conservation, we therefore opted for reconstituted human plasma as a surrogate. Synthetic peptides corresponding to γ2-MSH and the γ3-MSH variants depicted in Fig 6A were incubated in human plasma at 37°C. At the indicated time points, samples were taken and activity of peptide hormones detected *via* our functional receptor-signaling assay. The plasma half-life of owl-derived γ2-MSH was 27 min under our conditions (Fig 6A). The γ3-MSH variants had longer half-lives that gradually increased, proportional to the number of serine residues present (Fig 6A and 6B). The highest stability was observed with TA γ3-MSH containing 13 serine residues resulting in a half-life of 32 h, i.e. 70-fold longer than that of γ2-MSH and > 6-fold longer than that of γ3-MSH containing 5 serines (Fig 6A and 6B). Further extension of serine repeats to 18 and 19 residues decreased the plasma half-life of TA γ3-MSH to 19 and 14 h, respectively (Fig 6A and 6B). No unspecific signaling of γ-MSH or its plasma degradation products was observed (S7A Fig). In reconstituted human cerebrospinal fluid (CSF), both γ2-MSH and γ3-MSH peptides were stable for several hours (S7B Fig), likely due to reduced levels of degrading enzymes. The striking effect of the poly-serine length on the plasma stability of γ3-MSH suggests a role of the poly-serine polymorphism in the endocrine function of the peptide hormone.

### Stabilized γ3-MSH can compensate for loss of β-MSH signaling in *Tyto alba alba* (TA)

Our studies revealed that an increased length of the poly-serine stretch enhanced in particular plasma stability of TA γ3-MSH. We therefore hypothesized that increased bioavailability of γ3-MSH may somehow compensate for the loss of β-MSH signaling caused by the H9P mutation in the receptor-binding site. As mentioned above, we verified that POMC, PC1/3 and PC2 were expressed in the pituitary of TA, rendering the production of γ3-MSH as well as β-MSH and their release into the bloodstream feasible (S2 Fig). To test our hypothesis, we chose γ3-MSH with 10 serine residues as a representative example for a “stable” γ3-MSH form. As already shown in Fig 2G and 2H, β-MSH P9 was unable to compete with functional β-MSH H9 at the receptor level. However, we could not formally exclude the possibility that TA β-MSH P9 could somehow compete with γ-MSH in receptor signaling. We therefore monitored the MC3R and MC4R signaling activities of γ2-MSH and γ3-MSH with 10 serine residues in presence of increasing concentrations of TA β-MSH P9 (Fig 6D and 6E). Again, we observed no evident interference of β-MSH P9 with γ2-MSH or γ3-MSH receptor signaling (Fig 6D and 6E), opening the possibility that stabilized γ3-MSH carrying ≥7 serine residues could compensate for the defective β-MSH P9 in the periphery.

### The phenotype of the β-MSH H9P mutation and increased plasma stability of γ3-MSH *vs*. γ2-MSH are conserved in humans

Based on our findings in owl populations, we investigated possible conservation of observed POMC mutations and their phenotypes in humans. Searching of genetic databases revealed that the mutation H225P in human POMC, corresponding to the β-MSH H9P mutation, occurs at low frequency (S2 Table). In contrast to *Tyto* species, the human β-MSH H9P mutation occurs only heterozygous and is not associated with an overt phenotype (S2 Table). We next analyzed the impact of the human β-MSH H9P mutation on signaling through human MC3R and MC4R (Fig 6F and 6G). The H9P replacement in human β-MSH markedly reduced signaling activity on both receptors (>30 fold increased EC50), and lowered the signaling amplitude on MC3R (Fig 6F and 6G), similar to our findings in barn owls (Fig 2D–2F). The previously reported human β-MSH mutations Y5C and P15L, associated with obesity in heterozygous carriers [12, 14, 15], resulted in weaker effects in our hands (Fig 6H and 6I). The apparent discrepancy with the observed human phenotypes suggests effects of these mutations on the biosynthesis, folding, and processing of POMC. In analogy to the *Tyto* species, polymorphisms with extensions of the serine rich tail in γ3-MSH were identified in humans (S2 Table), in particular microsatellite amplifications of the SSG motif [11]. We therefore analyzed non-glycosylated synthetic peptides of these naturally occurring γ3-MSH human variants in MC3R and MC4R signaling. No major differences compared to wild-type human γ2- and γ3-MSH were observed (Fig 6J and 6K), similar to *Tyto alba alba* with serine extensions ≤13 residues (Fig 5A). Plasma stability of non-glycosylated human wild-type γ3-MSH was >100 fold increased over γ2-MSH (Fig 6L), closely matching the observed plasma stabilization in the barn owl. Moreover, addition of SSG motives further increased the half-life to >150 fold (Fig 6L). Although limited in scope, these studies suggest that the phenotype of the β-MSH H9P mutation and increased plasma stability of γ3-MSH vs. γ2-MSH may be conserved in humans.

## Discussion

**Fig 7:**
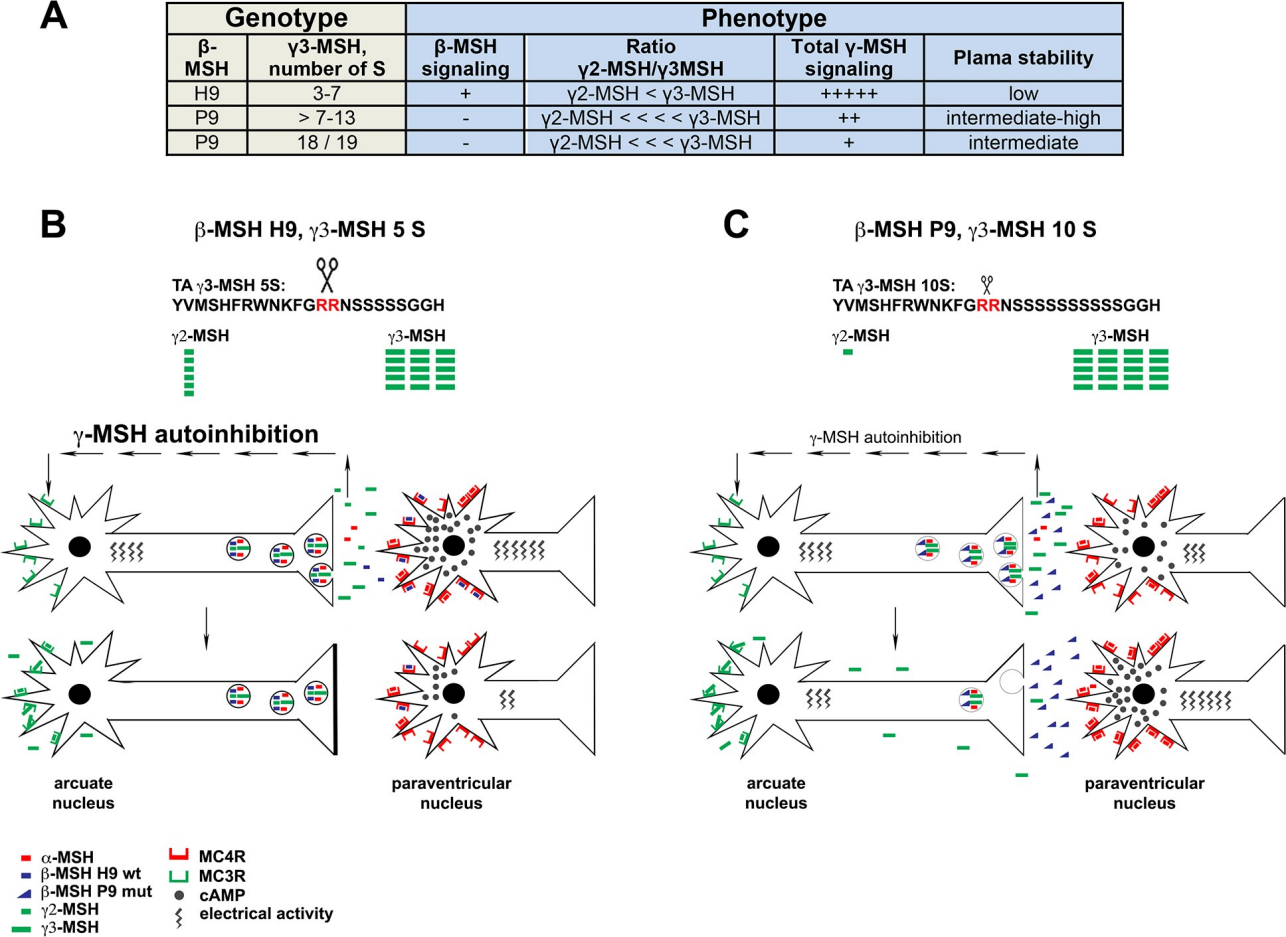
Model explaining central compensation of serine extended γ3-MSH for dysfunctional β-MSH P9 in the barn owl group. (A) Phenotypes associated with polymorphisms in β- and γ-MSH. (B, C) Working model: POMC and MC3R-expressing neurons in the arcuate nucleus of the hypothalamus projecting to MC4R expressing neurons of the paraventricular nucleus are shown. Anticipated MSH signaling scenarios are depicted for the combination of intact β-MSH H9 and γ3-MSH with < 7 serine residues (5 S) as in ancient clades of Tydonidae (B), and for dysfunctional β-MSH P9 and γ3-MSH with > 7 serine residues (10 S) as found in the barn owl group (C). The extension of serine residues in the POMC γ3-MSH locus to ≥7 increases the production of γ3-MSH at the cost of γ2-MSH, as exemplified by the γ3-MSH 10 S variant in the barn owl group (C). Since the MC3R signaling activity of γ3-MSH is reduced by approximately one order of magnitude compared to γ2-MSH, the auto-inhibitory γ-MSH activity on POMC expressing neurons of the arcuate nucleus is strongly decreased in C. As a consequence, the release of POMC derived MSH peptides is prolonged. The lack of β-MSH P9 signaling on post-synaptic MC4R in the paraventricular nucleus can therefore be compensated by increased amounts of α-MSH in the synaptic cleft. The elevated cAMP levels in neurons of the paraventricular nucleus resulting from MC4R activation can therefore be maintained and an energy imbalance prevented, despite dysfunctional β-MSH P9.

Our genetic studies in wild-living owl populations revealed a striking association between a H9P mutation in β-MSH and an extension of the poly-serine stretch in γ3-MSH to 7 to 24 residues in the Tytonidae family (Fig 7A). Phylogenetic analysis showed that only the barn owl group within the Tytonidae displayed the C**A**T to C**C**T single nucleotide polymorphism in β-MSH that resulted in a H9P mutation changing the receptor-binding site from **H**FRW to **P**FRW. In contrast, all other species analyzed within the Tytonidae carry β-MSH H9 alleles with intact **H**FRW receptor-binding sites in conjunction with γ3-MSH alleles bearing 4–7 serine residues. The closest relative of the barn owl group, the Madagascar red owl *Tyto soumagnei*, carries homozygous β-MSH H9 in combination with only one allelic variant of γ3-MSH containing 7 serine residues. According to our phylogenetic analysis, the Madagascar red owl and the barn owl group had a common ancestor around 8 MYA and the barn owl group diverged into distinct lineages approximately 6 MYA. The β-MSH P9 variant therefore likely emerged between 6 and 8 MYA in a common ancestor of the barn owl group, who may have already carried a γ-MSH variant with 7 serine residues (Fig 1).

Our functional studies with TA MCR orthologues revealed markedly reduced receptor signaling of β-MSH P9. This was not unexpected since H9 is part of the highly conserved HFRW receptor-binding motif common to α-, β- and γ-MSH and critical for MCR activation [42]. The phenotype of β-MSH P9 appears recessive, since β-MSH H9 receptor signaling was unaffected by a molar excess of β-MSH P9. In the barn owl group, including the Swiss population of TA, all 484 sequenced individuals were homozygous for β-MSH P9. Despite the absence of functional β-MSH in TA no overt signs and symptoms resembling the clinical manifestations of decreased functional levels of α- and /or β-MSH in human were found [11, 13–15, 43, 44]. We therefore hypothesized a compensatory effect by the poly-serine polymorphism in γ3-MSH.

First, we investigated the influence of the poly-serine repeat length in the γ3-MSH locus on POMC precursor processing by PC1/3 and PC2. We found that increased numbers of serine repeats in the γ3-MSH locus starting with a “threshold” of minimally 7 residues enhanced production of γ3-MSH at the expense of γ2-MSH. We further observed that cleavage of TA and SA POMC at the γ3-MSH internal site 2 arginine-arginine (RR) motif was rather inefficient. This might be due to shielding by the conserved N-glycan in position P2’ [31, 45], as described for cleavage by other basic PCs [46]. As a consequence, γ3-MSH but not γ2-MSH might represent the major γ-MSH form. The existence of γ2-MSH is however suggested in other species evidenced by its isolation e.g. from human heart and bovine pituitary [47, 48]. When comparing γ3-MSH with γ2-MSH, we discovered that γ3-MSH forms showed significantly diminished receptor signaling activity regardless of the number of serine residues, possibly due to steric hindrance of receptor-binding by the N-glycan in P2’.

Shifting production to a less active γ-MSH species in the absence of functional β-MSH, as observed in TA, seems at first sight puzzling. However, our observations might be explained in the context of an auto-inhibitory function of γ-MSH in the hypothalamic circuitry regulating food intake as suggested in our working model (Fig 7B and 7C). POMC expressing neurons in the arcuate nucleus of the hypothalamus project to the paraventricular nucleus where they release α-, β- and γ-MSH. MSH-mediated activation of MC4R on post-synaptic paraventricular neurons leads to suppression of appetite [6–8, 29]. Both α-MSH and β-MSH, but not γ-MSH reduced food intake at equimolar doses after intra-cerebral injection to fasted rats [7]. Loss-of-function mutations in human β-MSH are associated with obesity, despite the presence of functional α-MSH [12–15], suggesting that the overall dose of α- and β-MSH is critical for MC4R activation in the paraventricular nucleus. Notably, γ-MSH is implicated in MC3R-mediated auto-inhibition of the activity of POMC expressing neurons in the arcuate nucleus [8, 29, 49]. Here, we show that TA β-MSH P9 is dysfunctional. A shift towards production of γ-MSH forms with reduced signaling activity, as suggested by our studies for TA, may lead to prolonged MSH secretion from POMC-expressing neurons in the arcuate nucleus. The consequent increase of α-MSH levels in the paraventricular nucleus may compensate for the diminished post-synaptic MC4R activation due to defective β-MSH P9. Although speculative at this point, our model may explain at least in part the genetic link between β-MSH P9 and the serine extension to ≥7 residues in γ3-MSH of Tytonidae.

Our working model (Fig 7B and 7C) may provide an explanation for the increase in the number of serine residues in the TA POMC γ3-MSH locus from 7 to 10 with its accompanying shift from γ2- to γ3-MSH production, but not beyond, since no additional increase in the γ3-MSH steady state levels was observed from 10 to 18 serine residues. The driving force behind the observed extension >10 serine residues remained elusive. We therefore investigated the consequences of serine extension for the endocrine function of γ3-MSH. First, we confirmed the co-expression of POMC, PC1/3 and PC2 in the pituitary of TA, required for the release of *γ*3-MSH into the bloodstream. We then analyzed the plasma stability of *γ*3-MSH with varying serine repeat lengths. The plasma half-lives of synthetic γ3-MSH with ≥7 serine residues were prolonged compared to γ3-MSH with 3 or 5 serine residues. A maximal half-life of >30 h was reached with 13 serine residues, with a decline observed with 18 and 19 serine. Both β-MSH and γ3-MSH are implicated in lipolysis [50–52]. Stabilized γ3-MSH might thus compensate for impaired lipolysis caused by functionally deficient β-MSH P9. In addition, elevated γ3-MSH levels might be beneficial due to anti-inflammatory effects mediated through MC1R on neutrophils, monocytes and dendritic cells and MC3R on macrophages [53, 54]. Amplification to 18 and 19 serine residues in γ3-MSH seems suboptimal for plasma stability and peripheral signaling, but, due to increased EC50 on MC3R (Fig 5A), may further augment the efficiency of the central α-MSH compensation mechanism in the arcuate nucleus. In wild-living owls, these different compensation mechanisms may need to be balanced depending on environmental factors. This might be reflected in the shifted frequency distributions of γ3-MSH poly-serine lengths in *Tyto* species living in different habitats.

Intrigued by our findings in owl populations, we investigated possible conservation of the observed POMC mutations and their functional consequences in humans. The mutation H9P in β-MSH was found to occur in humans, albeit with low frequency and exclusively heterozygous. In analogy to our findings in the barn owl system, the H9P mutation resulted in a marked loss of biological function in human β-MSH. However, in contrast to the barn owl, known insertions of SSG motives in human γ3-MSH resulted in only incremental increases in plasma stability and no genetic linkage between the two polymorphisms has been reported in humans so far. This may also explain the currently strict heterozygosity of human β-MSH P9, suggesting a detrimental phenotype of homozygotes in absence of any compensation. Interestingly, a loss of function mutation of β-MSH found in Labrador dogs that does not affect the γ- and α-MSH loci leads to increased appetite and body weight [55]. This suggests a link between β-MSH deficiency and obesity in the absence of any compensation. Mice and rats also lack functional β-MSH, due to a deficient cleavage site. However, the simultaneous loss of the γ3-MSH internal dibasic cleavage site in these rodents yields exclusively γ3- but not γ2-MSH. Overall, this resembles the situation in *Tyto alba*, where homozygous loss of function in β-MSH is linked to increased γ3-over γ2-MSH production. Therefore the lack of any overt obesity phenotype in *Tyto alba* despite the homozygous H9P β-MSH loss of function mutation might be explained by compensation through serine amplification to 10–24 residues in the γ3-MSH locus, diminishing cleavage of POMC at site 2 and increasing the levels of γ3-MSH, in analogy to rodents. Likely, an initial γ3-MSH serine extension to a minimum of 7 residues was required for the homozygous H9P β-MSH mutation to be viable in *Tyto alba*, and was optimized by continued serine extension to further augment γ3-MSH levels. Lastly, a >100-fold stabilization in plasma can be observed for human γ3- compared to γ2-MSH, similar to the >70-fold stabilization in *Tyto alba*. This supports the notion that γ3-MSH might indeed represent the systemic or endocrine form of γ-MSH.

## Supporting information

S1 Fig *Tyto alba alba* (TA) and *Strix aluco* (SA) POMC polypeptide precursors are N-glycosylated. We analyzed in two naturally occurring POMC variants, TA POMC 18 S (A) and SA POMC 3 S (B), whether the number of serine residues influenced N-glycosylation at the neighboring asparagine residue. In lysates of HEK293T cells transfected with N-terminally HA-tagged *POMC*, three bands of POMC precursor could be detected by Western blot with anti-HA antibody for both species (mock treated lanes in A, B). Enzymatic treatment of lysates with both Peptide-N-Glycoidase F (PNGase F) and Endoglycosidase H (Endo H) resulted in disappearance of the high-mannose or hybrid structure, intermediary glycosylation product **(**intermediary band in A, B). Treatment of POMC with PNGase F additionally removed the fully matured, complex type oligosaccharide from the POMC protein backbone (highest band in A, B). Therefore, the asparagine residue next to the poly-serine stretch carries fully matured complex-type oligosaccharides in both, SA POMC 3 S and TA POMC 18 S, and N-glycosylation is unaffected by the large number of serine residues in TA.
(TIF)

S2 Fig Full length *POMC*, *PC1/3* and *PC2* are abundantly expressed in the pituitary and hypothalamus of *Tyto alba alba*. The relative expression levels of full length *POMC* (green bars), *PCSK1* (blue bars, coding for PC1/3), and *PCSK2* (red bars, coding for PC2) were determined by qPCR. Expression in all tissues was measured relative to the control genes Elongation factor 1A (*EEF1A*) and ribosomal protein L13 (*RPL13*), and subsequently normalized to expression in the pituitary, the tissue displaying the highest expression level for all three genes. Generally, expression of full length *POMC*, *PC1/3* and *PC2* was most pronounced in the brain. With relevance to the endocrine pathway, production of all POMC cleavage products, including γ3-MSH, should be feasible, given the expression of both proprotein convertases, PC1/3 and PC2 in the pituitary.
(TIF)

S3 Fig *In vitro* characterization of *Tyto alba alba* (TA) PC1/3 and PC2 activities. (A) HEK293T cells were transfected with plasmids coding for C-terminally myc-tagged TA PC1/3, or C-terminally myc-tagged TA PC2 + mouse 7B2, and PC expression was verified in cell lysates (L) and supernatants (S) by Western blot analysis using anti-myc antibody. (B-D) PC-containing supernatants or supernatants from mock-transfected cells (negative control) were tested *in vitro* for their cleavage efficiency of Pyr-ERTKR-AMC substrate. PC1/3 and PC2 cleavage activities were monitored as the increase of relative fluorescence units over time (RFU/min) during the phase of linear increase in signal intensity. (B) Within the analyzed pH range, TA PC1/3 displayed highest activity around pH 5, and PC2 in combination with 7B2 was most active at pH 4.5 to pH 6. No activity is observed in supernatants from mock-transfected cells across the entire pH range. (C) Both PC1/3 and PC2 are calcium-dependent as shown by the strong decrease in cleavage activity in the presence of 10 mM EDTA. (D) In contrast to TA PC1/3, TA PC2 requires the chaperone 7B2 for its activity. The activity of TA PC1/3 is unaffected by the presence of 7B2. The newly cloned TA proprotein convertases PC1/3 and PC2 thus exhibit the typical profile of their respective orthologues from other species such as mouse and human.
(TIF)

S4 Fig Identification of N-terminal POMC fragments in *Strix aluco* (SA) resulting from PC-cleavage. (A) Schematic representation of cloned N-terminal SA POMC fragments used as a size ladder in Western blot. All DNA constructs contain from N- to C-terminus: 1) The POMC signal peptide 2) the HA tag representing the new N-terminus in mature ladder fragments 3) the POMC sequence downstream of the signal peptide extending to PC cleavage sites 1, 2 or 3 with their dibasic cleavage motives RK, RR and KR in ladder fragments L1, L2 and L3 (3 serine residues), respectively. Additionally, fragments L2 and L3 lacking cleavage site 1 (L2 w/o cl1 and L3 w/o cl1) were produced by replacing the dibasic motive RK by EE to prevent potential trimming of these fragments to L1 by endogenous basic PCs. (B-E) HEK293T cells were transfected with one of the 5 ladder constructs depicted in A or with full length HA-tagged SA *POMC* and plasmids coding for TA PC1/3, PC2 and 7B2, to generate the full spectrum of SA POMC cleavage products (E). POMC and ladder fragments were detected in lysates and supernatants 84 h post transfection by Western blot using anti-HA antibody. (B, E) Fragments L2 and L2 w/o cl1 from cell lysates migrated at the same apparent molecular weight of 11 kDa, whereas fragment L1 was detected at 9 kDa, thus excluding L2 cleavage at site 1 by endogenous basic PCs. Fragments L3 and L3 w/o cl1 both produced identical patterns with two bands migrating at approximately 12 and 14 kDa, thus demonstrating the absence of cleavage at sites 1 and 2 by endogenous PCs. (D, F) Treatment of SA *or* TA POMC ladder fragment L3 with PNGase F resulted in a 2–3 kDa band shift identifying the upper band of L3 in lysates and supernatants in B, C, and E as the N-glycosylated form and the lower band as under-glycosylated form (3u) lacking the N-glycan. (C, E) The N-glycosylated form of the SA L3 ladder fragment represents the major form released into the supernatant. L1 is generally weakly expressed and cannot be detected in supernatants, neither can L2 w/o cl1 nor L3 w/o cl1 (C), likely due to misfolding and ER retention. (E) Side by side loading with SA ladder fragments identified the lowest band in supernatant of PC-digested SA POMC as cleavage site 2 product (11 kDa), whereas cleavage site 1 product was not detected. The faint band at ca. 12 kDa corresponded to the non N-glycosylated cleavage site 3 fragment (3u) and the more abundant ca. 14 kDa band to its N-glycosylated form **(**both, supernatants and lysates were detected on the same blot**)**.
(TIF)

S5 Fig Detection of PC1/3 and PC2 expression in comparative *Tyto alba alba* and *Strix aluco* POMC cleavage studies. HEK293T cell lysates corresponding to supernatants in Fig 3 were harvested 56 h post transfection and expression of myc-tagged PC1/3 (A) and PC2 (B) was confirmed in Western blot with myc antibody.
(TIF)

S6 Fig Detection of POMC, PC1/3 and PC2 in cell lysates from POMC cleavage time course. (A) Lysates of HEK293T cells transfected with SA or TA HA-tagged POMC variants in combination with plasmids coding for myc-tagged PC1/3, PC2 and 7B2 were harvested 104 h post transfection, the latest time point of supernatant sampling to establish POMC cleavage kinetics (Fig 4). POMC precursor was detected in lysates by Western blot using HA antibody. PC1/3 and PC2 expression was confirmed with anti-myc antibody. Anti-tubulin antibody was used to verify loading of comparable amounts of cells. (B) For each of the different POMC variants, cell lysates were loaded next to supernatants. Supernatants were taken from the earliest time point (28 h post transfection), which contains the highest proportion of uncleaved POMC (Fig 4B–4F). Only POMC with the fully matured, complex type N-glycan is secreted into the supernatant in all POMC variants tested (uppermost of the 3 bands in lysates).
(TIF)

S7 Fig The residual signaling activity of γ2-MSH and γ3-MSH in plasma is MCR specific and plasma stability decreased compared to CNS stability. (A) Residual γ2- and γ3-MSH signaling was determined after various incubation times in plasma (0 to 72 h) by measuring the cAMP response in HEK293T cells expressing TA MC3R and Glo-sensor. In parallel, the signaling activity of γ-MSH containing plasma samples was also tested on cells transfected with *Glo-sensor* and *IRES GFP* (black traces). In the presence of MC3R, the expected degradation curves were observed for SA / TA γ2-MSH, for SA γ3-MSH 3 S and for TA γ3-MSH with 5, 13 and 18 serine residues. None of these γ-MSH-containing aliquots yielded a signaling response above background in the absence of exogenously expressed MC3R. Thus, neither γ2- or γ3-MSH, nor intermediary γ-MSH breakdown products stimulate cAMP signaling via endogenously expressed cell surface receptors. (B) SA / TA γ2-MSH and TA γ3-MSH synthetic peptides with 3, 5, 13 and 18 S were diluted to 2 μM in human CSF and incubated at 37°C for various times up to 72 h. Residual γ-MSH activity was determined as described for plasma stability assays (Fig 6A). All γ3-MSH variants were completely stable in CSF and also γ2-MSH had a half-life > 7.5 h.
(TIF)

S1 Table Accession numbers of genes used in this paper. (DOCX)

S2 Table *Human* genetic variants of β- and γ-MSHs corresponding to *Tyto Alba* (TA) variants. (DOCX)

S3 Table Sample and provider names, localisation, year and sex of the samples used in this study. (XLSX)

S1 Data (DOCX)
