# Supplementary figures and images for: Molecular evolution of the proopiomelanocortin system in Barn owl species
Source: PLoS One. 2020 May 5;15(5):e0231163. doi: 10.1371/journal.pone.0231163 (PMC7199972; doi:10.1371/journal.pone.0231163)

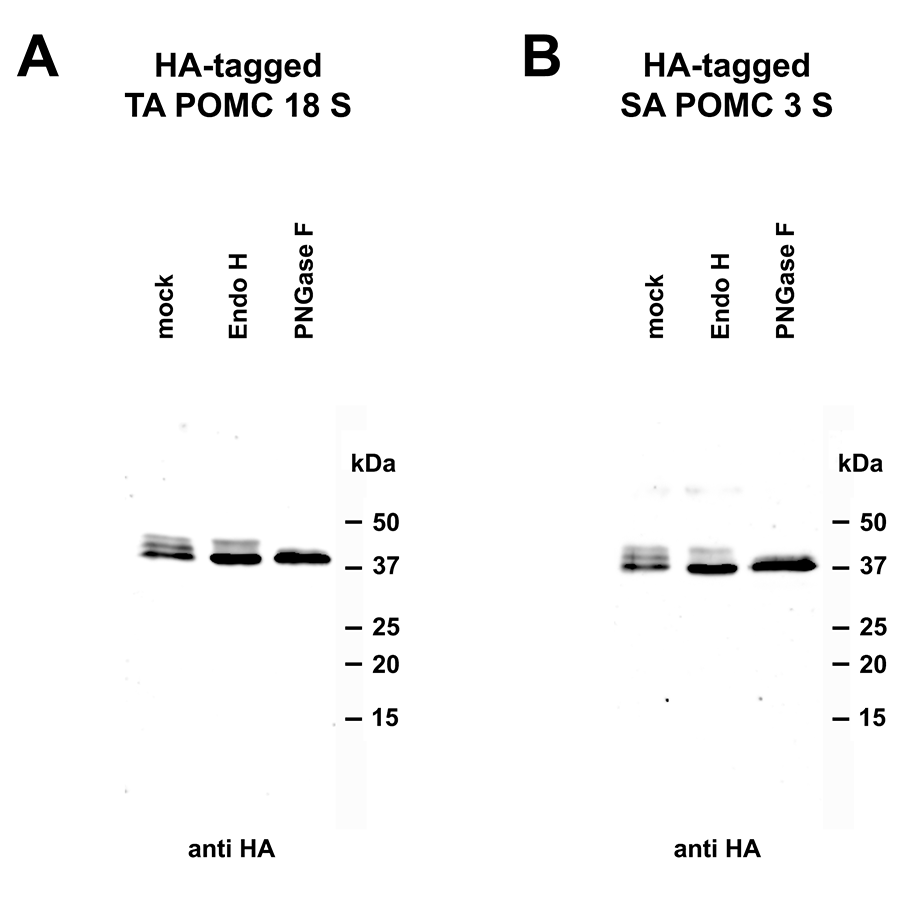

Supplement: S1 Fig — We analyzed in two naturally occurring POMC variants, TA POMC 18 S (A) and SA POMC 3 S (B), whether the number of serine residues influenced N-glycosylation at the neighboring asparagine residue. In lysates of HEK293T cells transfected with N-terminally HA-tagged POMC, three bands of POMC precursor could be detected by Western blot with anti-HA antibody for both species (mock treated lanes in A, B). Enzymatic treatment of lysates with both Peptide-N-Glycoidase F (PNGase F) and Endoglycosidase H (Endo H) resulted in disappearance of the high-mannose or hybrid structure, intermediary glycosylation product (intermediary band in A, B). Treatment of POMC with PNGase F additionally removed the fully matured, complex type oligosaccharide from the POMC protein backbone (highest band in A, B). Therefore, the asparagine residue next to the poly-serine stretch carries fully matured complex-type oligosaccharides in both, SA POMC 3 S and TA POMC 18 S, and N-glycosylation is unaffected by the large number of serine residues in TA. (TIF) [file pone.0231163.s001.tif]

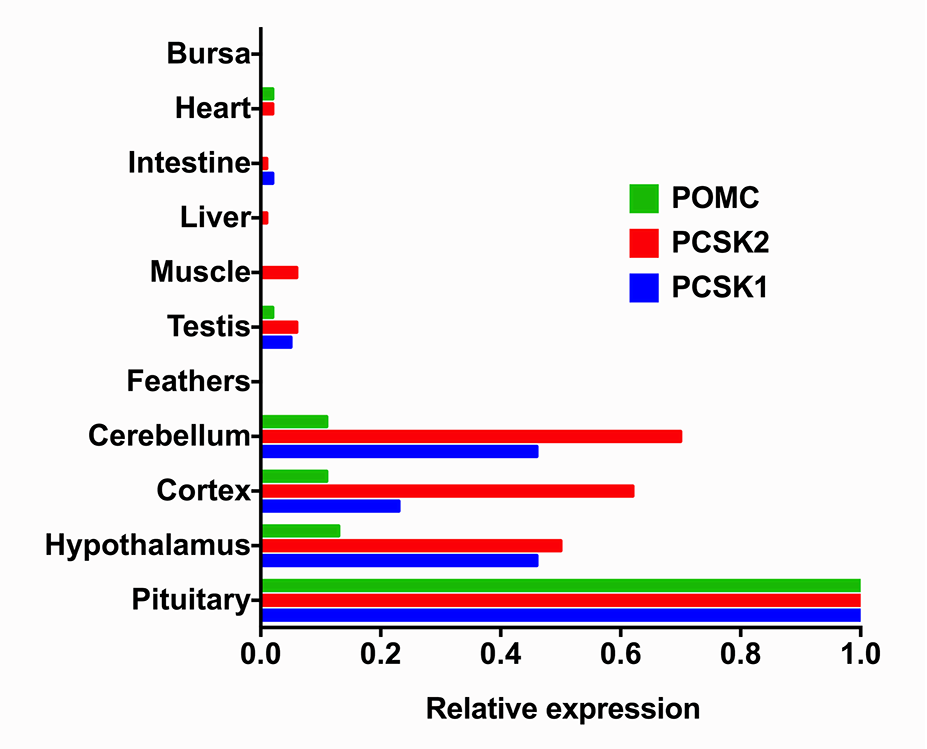

Supplement: S2 Fig — The relative expression levels of full length POMC (green bars), PCSK1 (blue bars, coding for PC1/3), and PCSK2 (red bars, coding for PC2) were determined by qPCR. Expression in all tissues was measured relative to the control genes Elongation factor 1A (EEF1A) and ribosomal protein L13 (RPL13), and subsequently normalized to expression in the pituitary, the tissue displaying the highest expression level for all three genes. Generally, expression of full length POMC, PC1/3 and PC2 was most pronounced in the brain. With relevance to the endocrine pathway, production of all POMC cleavage products, including γ3-MSH, should be feasible, given the expression of both proprotein convertases, PC1/3 and PC2 in the pituitary. (TIF) [file pone.0231163.s002.tif]

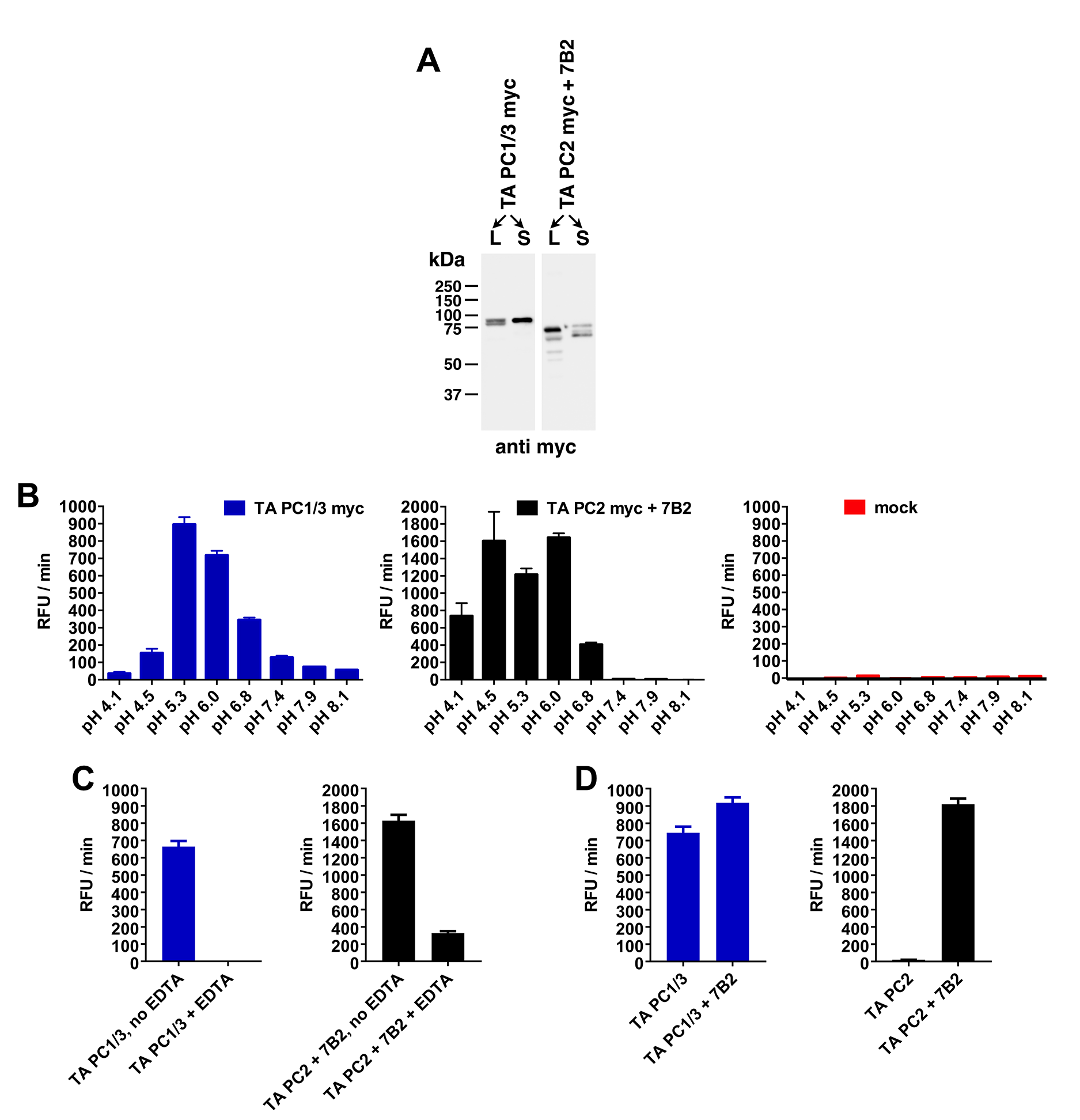

Supplement: S3 Fig — (A) HEK293T cells were transfected with plasmids coding for C-terminally myc-tagged TA PC1/3, or C-terminally myc-tagged TA PC2 + mouse 7B2, and PC expression was verified in cell lysates (L) and supernatants (S) by Western blot analysis using anti-myc antibody. (B-D) PC-containing supernatants or supernatants from mock-transfected cells (negative control) were tested in vitro for their cleavage efficiency of Pyr-ERTKR-AMC substrate. PC1/3 and PC2 cleavage activities were monitored as the increase of relative fluorescence units over time (RFU/min) during the phase of linear increase in signal intensity. (B) Within the analyzed pH range, TA PC1/3 displayed highest activity around pH 5, and PC2 in combination with 7B2 was most active at pH 4.5 to pH 6. No activity is observed in supernatants from mock-transfected cells across the entire pH range. (C) Both PC1/3 and PC2 are calcium-dependent as shown by the strong decrease in cleavage activity in the presence of 10 mM EDTA. (D) In contrast to TA PC1/3, TA PC2 requires the chaperone 7B2 for its activity. The activity of TA PC1/3 is unaffected by the presence of 7B2. The newly cloned TA proprotein convertases PC1/3 and PC2 thus exhibit the typical profile of their respective orthologues from other species such as mouse and human. (TIF) [file pone.0231163.s003.tif]

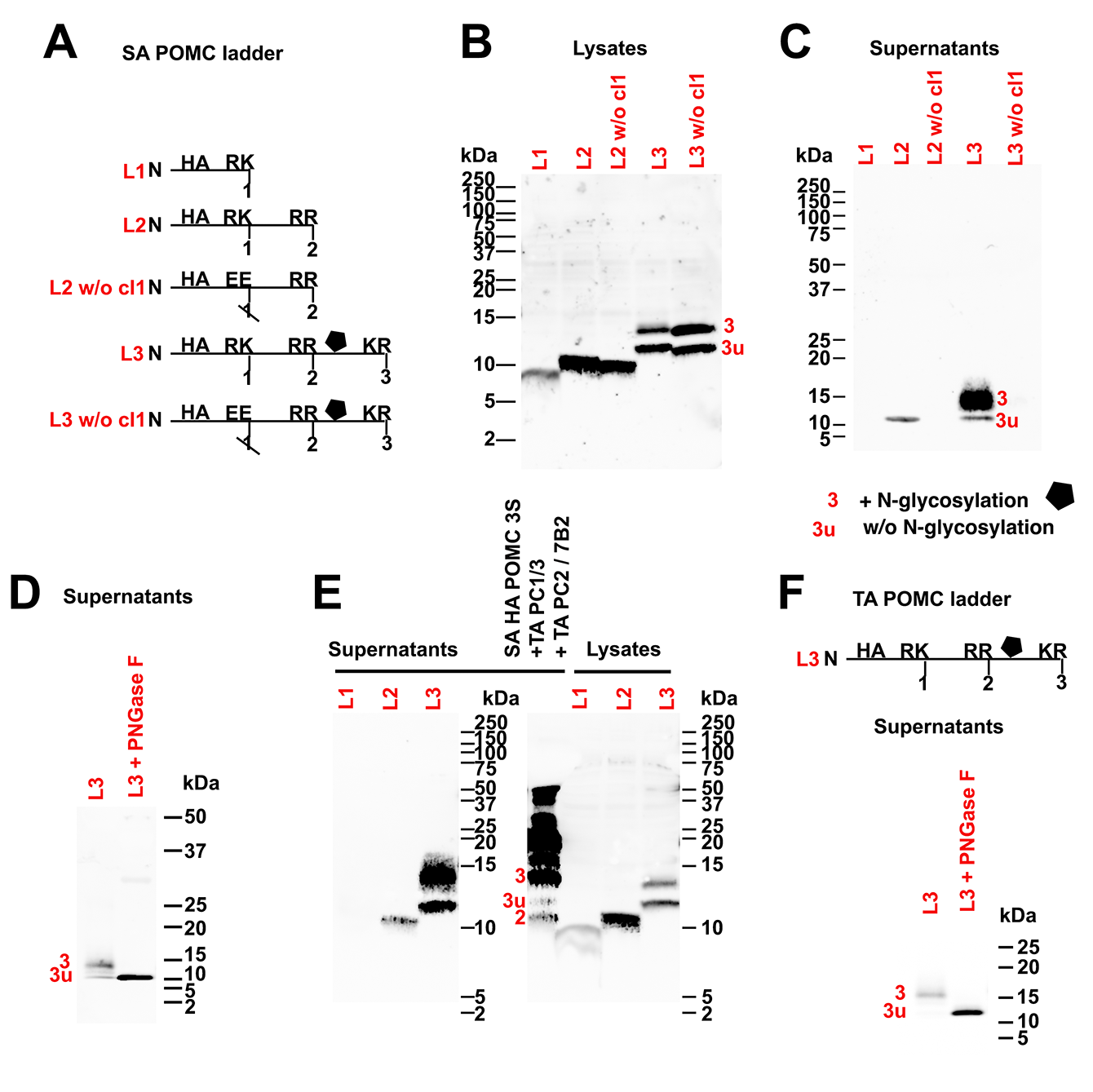

Supplement: S4 Fig — (A) Schematic representation of cloned N-terminal SA POMC fragments used as a size ladder in Western blot. All DNA constructs contain from N- to C-terminus: 1) The POMC signal peptide 2) the HA tag representing the new N-terminus in mature ladder fragments 3) the POMC sequence downstream of the signal peptide extending to PC cleavage sites 1, 2 or 3 with their dibasic cleavage motives RK, RR and KR in ladder fragments L1, L2 and L3 (3 serine residues), respectively. Additionally, fragments L2 and L3 lacking cleavage site 1 (L2 w/o cl1 and L3 w/o cl1) were produced by replacing the dibasic motive RK by EE to prevent potential trimming of these fragments to L1 by endogenous basic PCs. (B-E) HEK293T cells were transfected with one of the 5 ladder constructs depicted in A or with full length HA-tagged SA POMC and plasmids coding for TA PC1/3, PC2 and 7B2, to generate the full spectrum of SA POMC cleavage products (E). POMC and ladder fragments were detected in lysates and supernatants 84 h post transfection by Western blot using anti-HA antibody. (B, E) Fragments L2 and L2 w/o cl1 from cell lysates migrated at the same apparent molecular weight of 11 kDa, whereas fragment L1 was detected at 9 kDa, thus excluding L2 cleavage at site 1 by endogenous basic PCs. Fragments L3 and L3 w/o cl1 both produced identical patterns with two bands migrating at approximately 12 and 14 kDa, thus demonstrating the absence of cleavage at sites 1 and 2 by endogenous PCs. (D, F) Treatment of SA or TA POMC ladder fragment L3 with PNGase F resulted in a 2–3 kDa band shift identifying the upper band of L3 in lysates and supernatants in B, C, and E as the N-glycosylated form and the lower band as under-glycosylated form (3u) lacking the N-glycan. (C, E) The N-glycosylated form of the SA L3 ladder fragment represents the major form released into the supernatant. L1 is generally weakly expressed and cannot be detected in supernatants, neither can L2 w/o cl1 nor L3 w/o cl1 (C), likely due to misfo [file pone.0231163.s004.tif]

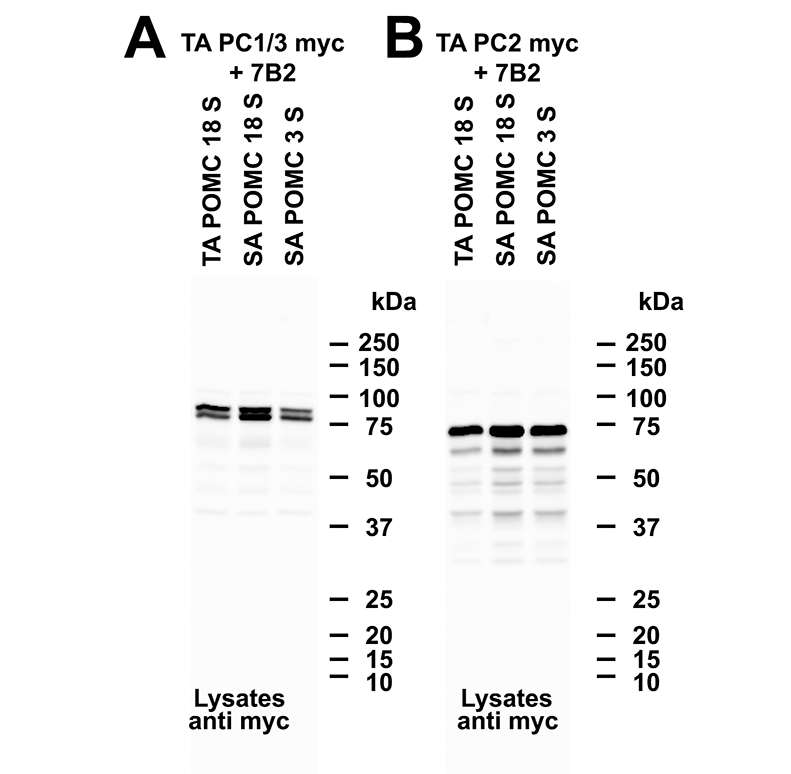

Supplement: S5 Fig — HEK293T cell lysates corresponding to supernatants in Fig 3 were harvested 56 h post transfection and expression of myc-tagged PC1/3 (A) and PC2 (B) was confirmed in Western blot with myc antibody. (TIF) [file pone.0231163.s005.tif]

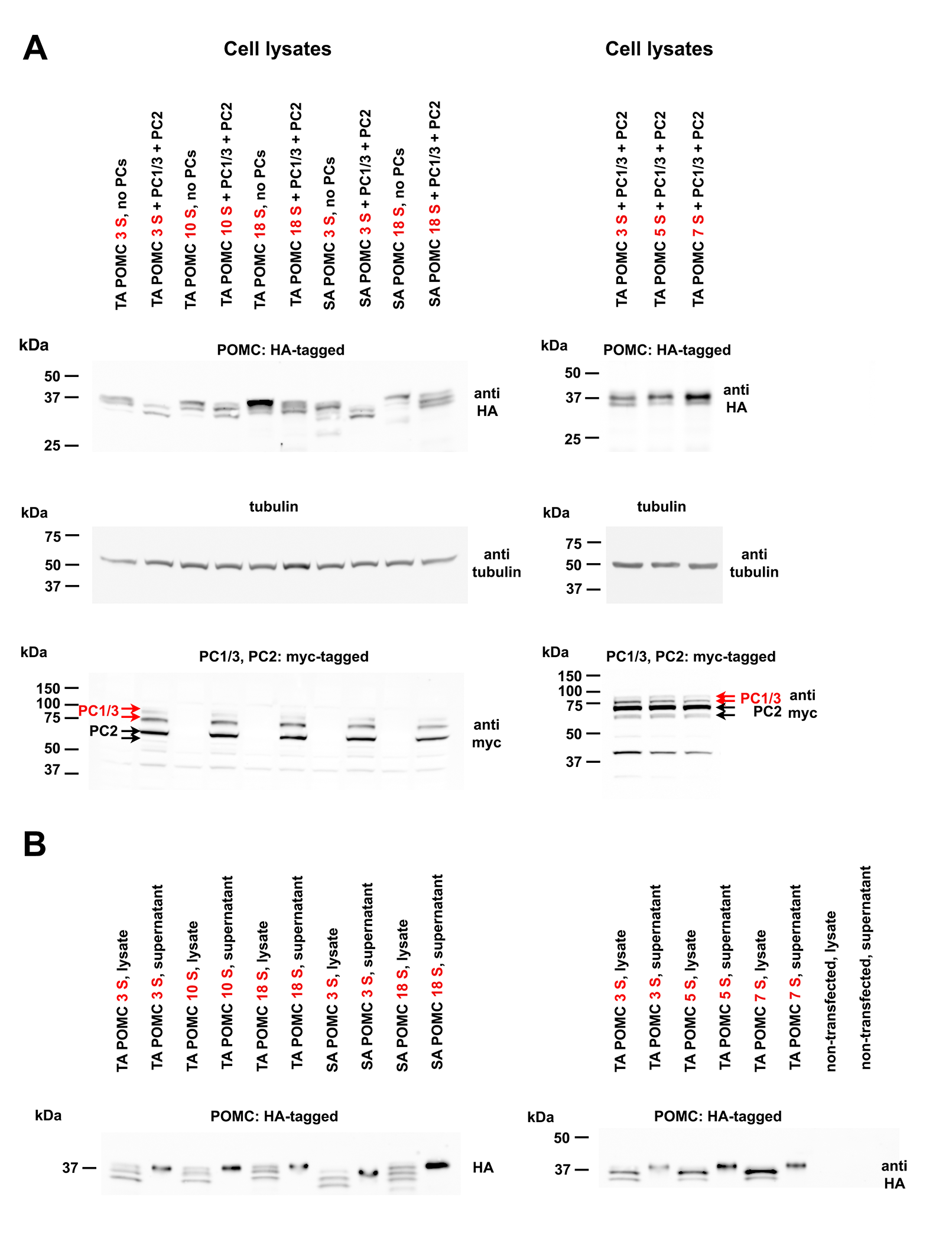

Supplement: S6 Fig — (A) Lysates of HEK293T cells transfected with SA or TA HA-tagged POMC variants in combination with plasmids coding for myc-tagged PC1/3, PC2 and 7B2 were harvested 104 h post transfection, the latest time point of supernatant sampling to establish POMC cleavage kinetics (Fig 4). POMC precursor was detected in lysates by Western blot using HA antibody. PC1/3 and PC2 expression was confirmed with anti-myc antibody. Anti-tubulin antibody was used to verify loading of comparable amounts of cells. (B) For each of the different POMC variants, cell lysates were loaded next to supernatants. Supernatants were taken from the earliest time point (28 h post transfection), which contains the highest proportion of uncleaved POMC (Fig 4B–4F). Only POMC with the fully matured, complex type N-glycan is secreted into the supernatant in all POMC variants tested (uppermost of the 3 bands in lysates). (TIF) [file pone.0231163.s006.tif]

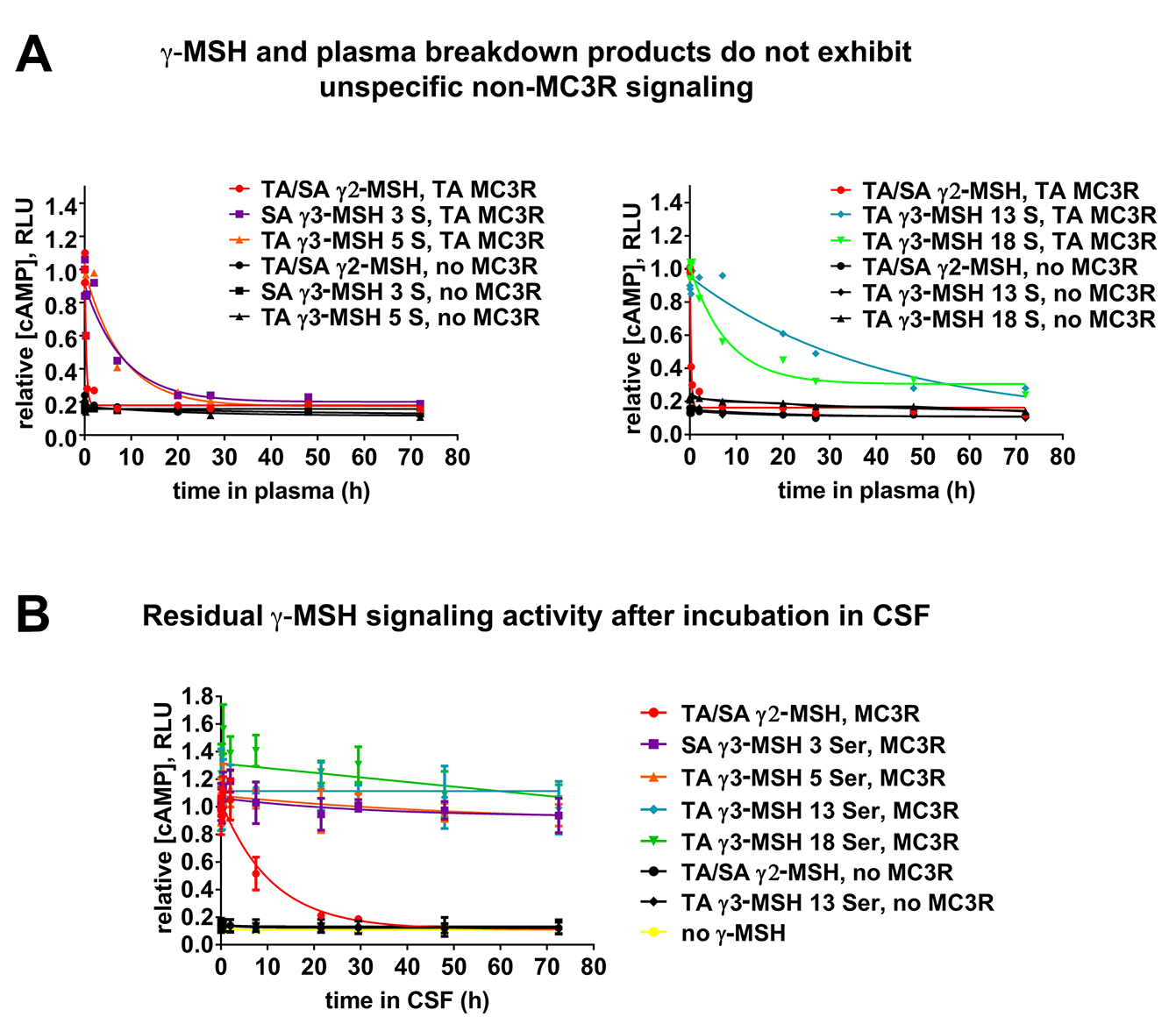

Supplement: S7 Fig — (A) Residual γ2- and γ3-MSH signaling was determined after various incubation times in plasma (0 to 72 h) by measuring the cAMP response in HEK293T cells expressing TA MC3R and Glo-sensor. In parallel, the signaling activity of γ-MSH containing plasma samples was also tested on cells transfected with Glo-sensor and IRES GFP (black traces). In the presence of MC3R, the expected degradation curves were observed for SA / TA γ2-MSH, for SA γ3-MSH 3 S and for TA γ3-MSH with 5, 13 and 18 serine residues. None of these γ-MSH-containing aliquots yielded a signaling response above background in the absence of exogenously expressed MC3R. Thus, neither γ2- or γ3-MSH, nor intermediary γ-MSH breakdown products stimulate cAMP signaling via endogenously expressed cell surface receptors. (B) SA / TA γ2-MSH and TA γ3-MSH synthetic peptides with 3, 5, 13 and 18 S were diluted to 2 μM in human CSF and incubated at 37°C for various times up to 72 h. Residual γ-MSH activity was determined as described for plasma stability assays (Fig 6A). All γ3-MSH variants were completely stable in CSF and also γ2-MSH had a half-life > 7.5 h. (TIF) [file pone.0231163.s007.tif]
