# Supplementary material for: Molecular evolution of the proopiomelanocortin system in Barn owl species
Source: PLoS One. 2020 May 5;15(5):e0231163. doi: 10.1371/journal.pone.0231163 (PMC7199972; doi:10.1371/journal.pone.0231163)
Supplement: S1 Table — (DOCX) [file pone.0231163.s008.docx]

**S1 Table: Accession numbers of genes used in this paper**

| **Genes** | **Species** | **Accession number** |
| --- | --- | --- |
| MC3R | *Tyto alba alba* | KY189305 |
| MC4R | *T. alba alba* | KY189307-8 |
| PCSK1 | *T. alba alba* | KU712308-9 |
| PCSK2 | *T. alba alba* | KU712310-11 |
| POMC | *T. alba alba* | MG711331 |
| POMC | *T. alba alba* | MG711332 |
| POMC | *T. alba alba* | MG711333 |
| POMC | *T. alba alba* | MG711334 |
| POMC | *T. alba alba* | MG711335 |
| POMC | *T. alba alba* | MG711336 |
| POMC | *T. alba alba* | MG711337 |
| POMC | *T. furcata pratincola* | MG711338 |
| POMC | *T. furcata pratincola* | MG711339 |
| POMC | *T. furcata pratincola* | MG711340 |
| POMC | *T. furcata pratincola* | MG711341 |
| POMC | *T. furcata pratincola* | MG711342 |
| POMC | *T. javanica delicatula* | MG711343 |
| POMC | *T. javanica delicatula* | MG711344 |
| POMC | *T. javanica delicatula* | MG711345 |
| POMC | *T. javanica javanica* | MG711346 |
| POMC | *T. javanica javanica* | MG711347 |
| POMC | *T. javanica javanica* | MG711348 |
| POMC | *T. javanica javanica* | MG711349 |
| POMC | *T. alba erlangeri* | MG711350 |
| POMC | *T. alba erlangeri* | MG711351 |
| POMC | *T. furcata lucayana* | MG711352 |
| POMC | *T. furcata lucayana* | MG711353 |
| POMC | *T. alba affinis* | MG711354 |
| POMC | *T. alba affinis* | MG711355 |
| POMC | *T. alba affinis* | MG711356 |
| POMC | *T. alba affinis* | MG711357 |
| POMC | *T. alba affinis* | MG711358 |
| POMC | *T. alba affinis* | MG711359 |
| POMC | *T. alba affinis* | MG711360 |
| POMC | *T. alba affinis* | MG711361 |
| POMC | *T. alba affinis* | MG711362 |
| POMC | *T. alba affinis* | MG711363 |
| POMC | *T. alba ernesti* | MG711364 |
| POMC | *T. furcata furcata* | MG711365 |
| POMC | *T. furcata furcata* | MG711366 |
| POMC | *T. furcata furcata* | MG711367 |
| POMC | *T. tenebricosa arfaki* | MG711368 |
| POMC | *T. tenebricosa arfaki* | MG711369 |
| POMC | *T. tenebricosa multipunctata* | MG711370 |
| POMC | *T. tenebricosa multipunctata* | MG711371 |
| POMC | *T. tenebricosa tenebricosa* | MG711372 |
| POMC | *T. tenebricosa tenebricosa* | MG711373 |
| POMC | *T. tenebricosa multipunctata* | MG711374 |
| POMC | *T. soumagnei* | MG711375 |
| POMC | *T. novaehollandiae castanops* | MG711376 |
| POMC | *T. novaehollandiae castanops* | MG711377 |
| POMC | *T. novaehollandiae castanops* | MG711378 |
| POMC | *T. novaehollandiae castanops* | MG711379 |
| POMC | *T. novaehollandiae novaehollandiae* | MG711380 |
| POMC | *T. novaehollandiae novaehollandiae* | MG711381 |
| POMC | *T. novaehollandiae novaehollandiae* | MG711382 |
| POMC | *T. longimembris Asia* | MG711383 |
| POMC | *T. longimembris Asia* | MG711384 |
| POMC | *T. longimembris Asia* | MG711385 |
| POMC | *T. longimembris walleri* | MG711386 |
| POMC | *T. longimembris walleri* | MG711387 |
| POMC | *T. longimembris longimembris* | MG711388 |
| POMC | *T. longimembris longimembris* | MG711389 |
| POMC | *T. longimembris amauronota* | MG711390 |
| POMC | *T. longimembris longimembris* | MG711391 |
| POMC | *T. longimembris amauronota* | MG711392 |
| POMC | *T. capensis* | MG711393 |
| POMC | *T. capensis* | MG711394 |
| POMC | *T. capensis* | MG711395 |
| POMC | *Phodilus badius* | MG711396 |
| POMC | *Phodilus badius* | MG711397 |
| POMC | *T. alba detorta* | MG711398 |
| POMC | *T. alba detorta* | MG711399 |
| POMC | *T. alba detorta* | MG711400 |
| POMC | *T. alba rosenbergii* | MG711401 |
| POMC | *T. alba rosenbergii* | MG711402 |
| POMC | *T. alba rosenbergii* | MG711403 |
| POMC | *T. javanica lifuensis* | MG711404 |
| POMC | *T. javanica lifuensis* | MG711405 |
| POMC | *T. javanica lifuensis* | MG711406 |
| POMC | *T. javanica lulu* | MG711407 |
| POMC | *T. javanica lulu* | MG711408 |
| POMC | *T. javanica submaensis* | MG711409 |
| POMC | *T. alba hypermetra* | MG711410 |
| POMC | *T. alba hypermetra* | MG711411 |
| POMC | *T. alba hypermetra* | MG711412 |
| POMC | *T. alba hypermetra* | MG711413 |
| POMC | *T. alba hypermetra* | MG711414 |
| POMC | *T. alba hypermetra* | MG711415 |
| POMC | *T. furcata contempta* | MG711416 |
| POMC | *T. furcata contempta* | MG711417 |
| POMC | *T. furcata contempta* | MG711418 |
| POMC | *T. furcata tuidara* | MG711419 |
| POMC | *T. furcata guatemalae* | MG711420 |
| POMC | *T. furcata guatemalae* | MG711421 |
| POMC | *T. furcata tuidara* | MG711422 |
| POMC | *T. furcata tuidara* | MG711423 |
| POMC | *T. furcata tuidara* | MG711424 |
| POMC | *T. furcata insularis* | MG711425 |
| POMC | *T. furcata insularis* | MG711426 |
| POMC | *T. alba gracilirostris* | MG711427 |
| POMC | *T. alba thomensis* | MG711428 |
| POMC | *T. alba thomensis* | MG711429 |
| POMC | *T. furcata nigrescens* | MG711430 |
| POMC | *T. furcata bargei* | MG711431 |
| POMC | *T. furcata bargei* | MG711432 |
| POMC | *T. furcata bargei* | MG711433 |
| POMC | *T. furcata bargei* | MG711434 |
| POMC | *T. furcata punctatissima* | MG711435 |
| POMC | *T. furcata punctatissima* | MG711436 |
| POMC | *T. furcata punctatissima* | MG711437 |
| POMC | *T. glaucops* | MG711438 |
| POMC | *T. glaucops* | MG711439 |
| POMC | *T. javancia interposita* | MG711440 |
| POMC | *T. javancia interposita* | MG711441 |
| POMC | *T. javanica delicatula* | MG711442 |
| POMC | *T. nigrobrunnea* | MG711443 |
| POMC | *T. nigrobrunnea* | MG711444 |
| POMC | *T. nigrobrunnea* | MG711445 |
| POMC | *T. aurantia* | MG711446 |
| POMC | *T. aurantia* | MG711447 |
| POMC | *T. almae* | MG711448 |
| POMC | *T. inexspectata* | MG711449 |
| POMC | *T. inexspectata* | MG711450 |
| POMC | *Strix aluco* | KF201581 |
