# Supplementary material for: Molecular evolution of the proopiomelanocortin system in Barn owl species
Source: PLoS One. 2020 May 5;15(5):e0231163. doi: 10.1371/journal.pone.0231163 (PMC7199972; doi:10.1371/journal.pone.0231163)
Supplement: S2 Table — (DOCX) [file pone.0231163.s009.docx]

**S2 Table. *Human* genetic variants of β- and γ-MSHs corresponding to *Tyto alba* (TA) variants.**

| **Protein variant** | **Variant**  **location** | **Nucleotide**  **variant** | **Variant type** | **Variant allele count** | **Total**  **Allele**  **number** | **Homoz.allele number** | **Allele frequency** | **Clinvar** | **Described phenotypes** |
| --- | --- | --- | --- | --- | --- | --- | --- | --- | --- |
| TA POMC  p.S93_S94  ins 10 to 24 S | γ_3_MSH | c.280_309  to 351  dup(AGC) | in-frame insertion | 11704 | 11704 | See Figure 1 | 1 | this study | this study |
| Human POMC  p.S97_G99  del  SSG | 2:25384456 (rs10654394) | c.289_297delAGCAGCGGC | in-frame deletion | 3 | 18130 | 0 | 0.000166 | likely benign | ND |
| Human POMC  p.S97_G99  dup  SSG | 2:25384456 (rs10654394) | c.289_297  dupAGCAGCGGC | in-frame insertion | 1047 | 18130 | 53 | 0.057750 | likely benign | Same frequency in obese, healthy under-weight and anorexic groups (1), associated with an increase in serum leptin levels (2, 3), and fasting insulin levels in heterozygotes compared to the wild-type homozygotes (frequency of the mutation: 0.053 in obese and 0.045 in healthy (4), no effect on BMI (3, 5). |
| Human POMC  p.S94_G99  dup  SSGSSG | 2:25384456 (rs10654394) | c.280_297  dupAGCAGCGGCAGCAGCGGC | in-frame insertion | 8 | 18130 | 0 | 0.000441 | likely benign | SSG SSG insertion mutant only detected in 1 heterozygote obese female, but not in 60 underweight and 46 anorexic controls (1). |
| Human POMC  p.G99_A100  ins  SSGSSGSSGSSG | 2:25384456 (rs10654394) | c.297_298  ins(AGCAGCGGCAGCAGCGGC)2 | in-frame insertion | 1 | 18130 | 0 | 0.000055 | likely benign | ND |
| TA β-MSH  p.Y5H | β-MSH | c.626C>T | missense | 33 | 272 | 1 | 0.121324 | this study | this study |
| Human POMC  p.Y221C | [2:25384092 (rs149540566)](http://gnomad.broadinstitute.org/variant/2-25384092-T-C) | c.662A>G | missense | 94 | 118046 | 0 | 0.000796 | ND | Heterozygous Y221C mutation found in 5, 2, 1 out of 538, 722 and 485 obese patients, respectively (6-8) and only in 4/5152 non-obese Caucasian controls (7). |
| TA β-MSH  p.M7V | β-MSH | c.632A>G | missense | 272 | 272 | 136 | 1 | this study | this study |
| Human POMC  p.M223V | [2:25384087 (rs769309229)](http://gnomad.broadinstitute.org/variant/2-25384087-T-C) | c.667A>G | missense | 1 | 118730 | 0 | 0.000008 | ND | ND |
| Human POMC  p.M223T | [2:25384086 (rs150343979)](http://exac.broadinstitute.org/variant/2-25384086-A-G) | c.668T>C | missense | 4 | 118848 | 0 | 0.000034 | ND | ND |
| TA β-MSH  p.H9P | β-MSH active site | c.639A>T | missense | 272 | 272 | 136 | 1 | this study | this study |
| Human POMC  p.H225P | [2:25384080 (rs776328489)](http://gnomad.broadinstitute.org/variant/2-25384080-T-G) | c.674A>C | missense | 2 | 119166 | 0 | 0.000017 | ND | ND |
| Human POMC  p.H225Q | [2:25384079 (rs769179230)](http://gnomad.broadinstitute.org/variant/2-25384079-G-C) | c.675C>G | missense | 2 | 119352 | 0 | 0.000017 | ND | ND |

Homoz.= homozygous; p. = protein/peptide position; c. = cDNA position; dup = duplication; del = deletion; Human allele numbers are from <http://exac.broadinstitute.org>, ND : not described

**REFERENCES**

1. Hinney A, Becker I, Heibult O, Nottebom K, Schmidt A, Ziegler A, et al. Systematic mutation screening of the pro-opiomelanocortin gene: identification of several genetic variants including three different insertions, one nonsense and two missense point mutations in probands of different weight extremes. J Clin Endocrinol Metab. 1998;83(10):3737-41.

2. Miraglia del Giudice E, Cirillo G, Santoro N, D'Urso L, Carbone MT, Di Toro R, et al. Molecular screening of the proopiomelanocortin (POMC ) gene in Italian obese children: report of three new mutations. International journal of obesity and related metabolic disorders : journal of the International Association for the Study of Obesity. 2001;25(1):61-7.

3. Rosmond R, Ukkola O, Bouchard C, Bjorntorp P. Polymorphisms in exon 3 of the proopiomelanocortin gene in relation to serum leptin, salivary cortisol, and obesity in Swedish men. Metabolism. 2002;51(5):642-4. Epub 2002/04/30.

4. Santoro N, del Giudice EM, Cirillo G, Raimondo P, Corsi I, Amato A, et al. An insertional polymorphism of the proopiomelanocortin gene is associated with fasting insulin levels in childhood obesity. J Clin Endocrinol Metab. 2004;89(10):4846-9.

5. Wang F, Gelernter J, Kranzler HR, Zhang H. Identification of POMC exonic variants associated with substance dependence and body mass index. PLoS ONE. 2012;7(9):e45300. Epub 2012/10/03.

6. Biebermann H, Castaneda TR, van Landeghem F, von Deimling A, Escher F, Brabant G, et al. A role for beta-melanocyte-stimulating hormone in human body-weight regulation. Cell Metab. 2006;3(2):141-6.

7. Lee YS, Challis BG, Thompson DA, Yeo GS, Keogh JM, Madonna ME, et al. A POMC variant implicates beta-melanocyte-stimulating hormone in the control of human energy balance. Cell Metab. 2006;3(2):135-40.

8. Nordang GBN, Busk OL, Tveten K, Hanevik HI, Fell AKM, Hjelmesaeth J, et al. Next-generation sequencing of the monogenic obesity genes LEP, LEPR, MC4R, PCSK1 and POMC in a Norwegian cohort of patients with morbid obesity and normal weight controls. Mol Genet Metab. 2017;121(1):51-6.
