# Supplementary material for: Molecular evolution of the proopiomelanocortin system in Barn owl species
Source: PLoS One. 2020 May 5;15(5):e0231163. doi: 10.1371/journal.pone.0231163 (PMC7199972; doi:10.1371/journal.pone.0231163)
Supplement: S1 Data — (DOCX) [file pone.0231163.s011.docx]

**Molecular evolution of the proopiomelanocortin system in Barn owl species**

Karin Löw^1,2^, Anne-Lyse Ducrest^1^, Luis M. San-Jose^1^, Céline Simon^1^, Vera Uva^1^, Nabil G. Seidah^3^, Antonella Pasquato^2,4^, Stefan Kunz^2,4,5*^, and Alexandre Roulin^1,4*^

**Supporting results**

**The poly-serine polymorphism does not affect POMC N-glycosylation**

SA and TA POMC pro-hormones contain a unique N-glycosylation motif NXS/T (1, 2). Fully glycosylated forms of POMC represent the actual substrate processed by the proprotein convertases within the secretory pathway (3). We therefore investigated the effect of the poly-serine polymorphism on the extent of POMC N-glycosylation and secretion. To this end, we compared POMC from SA containing 3 serine residues with POMC from TA with 18 serine residues. Each construct was tagged with HA and V5 at the N- and C-termini, respectively. When expressed in mammalian HEK293T cells and resolved by SDS-PAGE, both POMC variants appeared as three distinct bands in cell lysates (S1A and S1B Figs). As expected, mainly the fully glycosylated POMC was secreted (S6B Fig). Treatment of cell lysates with the broadly specific N-glycosidase PNGase F resulted in loss of the two higher molecular mass bands, verifying the presence of N-glycans among POMC forms of both owl species (S1A and S1B Figs). When glycoproteins exit from the ER, the immature mannose N-glycans undergo further extension in the Golgi and acquire resistance to the glycosidase Endo H. Treatment of owl-derived POMC with Endo H in cell lysates resulted in selective loss of the intermediate band, indicating the presence of high mannose or hybrid N-glycans in both owl species (S1A and S1B Figs). In sum, the data reveal that SA and TA POMC undergo N-glycosylation and that the fully glycosylated, complex form is secreted. However, the extent of N-glycosylation seems neither affected by the species, nor the number of flanking serine repeats.

**Identification of PC1/3- and PC2-specific cleavage sites in *Tyto alba alba* and *Strix aluco* POMC**

To define the identity of POMC cleavage products, we generated a panel of SA POMC fragments reaching from the N-terminus to cleavage sites 1, 2, or 3 (S4A Fig). The individual N-terminal SA POMC fragments were then used as markers to identify the exact fragments generated by digestion of full-length SA POMC with PC1/3 and PC2 simultaneously (S4E Fig). The smallest detectable, PC-cleaved N-terminal POMC fragment in SA was identified as cleavage site 2 product. In fact cleavage site 1 product was never found in supernatants (S4E Fig). Also, the ladder fragment L1 was not detected in supernatants and showed only faint expression in cell lysates (S4B, S4C and S4E Figs). The SA POMC ladder fragment L3 displayed a double band, with the lower band representing only a minor fraction in cell supernatants (S4B, S4C and S4E Figs). PNGase F treatment revealed that ladder fragment L3 was N-glycosylated, just as full length POMC precursor, and allowed to assign a molecular weight of approximately 2-3 kDa to the N-glycan in SA (S4D Fig) and TA (S4F Fig). Thus, the major form of secreted SA ladder fragment L3 was N-glycosylated and only a small fraction was underglycosylated as also observed for the N-terminal cleavage site 3 fragment of PC-digested, SA POMC (band 3u, S4C and S4E Figs). Release of incompletely glycosylated cleavage site 3 fragment is likely due to ER overloading in the over-expression setting and escape of a small proportion of POMC from high mannose core oligosacharide transfer in the endoplasmic reticulum. Having identified the smallest visible N-terminal POMC cleavage product in SA, and having established the molecular weight of the N-glycans in SA and TA, we were able to predict the apparent molecular mass of all possible PC-generated, N-terminal POMC cleavage fragments based on their amino acid composition (Fig 3A).

**Human genetic database searches**

Human genetic data searches included the following databases:

GnomAD browser beta; <http://gnomad.broadinstitute.org>

Exom Aggregation Consortium <http://exac.broadinstitute.org>; <https://www.ncbi.nlm.nih.gov/snp>).

**References**

1. Siciliano RA, Morris HR, McDowell RA, Azadi P, Rogers ME, Bennett HP, et al. The Lewis x epitope is a major non-reducing structure in the sulphated N-glycans attached to Asn-65 of bovine pro-opiomelanocortin. Glycobiology. 1993;3(3):225-39.

2. Seidah NG, Rochemont J, Hamelin J, Lis M, Chretien M. Primary structure of the major human pituitary pro-opiomelanocortin NH2-terminal glycopeptide. Evidence for an aldosterone-stimulating activity. The Journal of biological chemistry. 1981;256(15):7977-84. Epub 1981/08/10.

3. Phillips MA, Budarf ML, Herbert E. Glycosylation events in the processing and secretion of pro-ACTH-endorphin in mouse pituitary tumor cells. Biochemistry. 1981;20(6):1666-75.
